# Development of new tools to study membrane-anchored mammalian Atg8 proteins

**DOI:** 10.1080/15548627.2022.2132040

**Published:** 2022-10-17

**Authors:** Sang-Won Park, Pureum Jeon, Akinori Yamasaki, Hye Eun Lee, Haneul Choi, Ji Young Mun, Yong-Woo Jun, Ju-Hui Park, Seung-Hwan Lee, Soo-Kyeong Lee, You-Kyung Lee, Hyun Kyu Song, Michael Lazarou, Dong-Hyong Cho, Masaaki Komatsu, Nobuo N. Noda, Deok-Jin Jang, Jin-A Lee

**Affiliations:** a Department of Vector Entomology, College of Ecology and Environment, Kyungpook National University, Sangju, Korea; b Department of Biological Sciences and Biotechnology, College of Life Sciences and Nanotechnology, Hannam University, Daejeon, Korea; c Institute of Microbial Chemistry, Tokyo, Japan; d Neural circuit research group, Korea Brain Research Institute, Daegu, Korea; e Department of Ecological Science, College of Ecology and Environment, Kyungpook National University, Sangju, Korea; f Department of Life Sciences, Korea University, Seoul, Korea; g Department of Biochemistry and Molecular Biology, Biomedicine Discovery Institute, Monash University, Victoria, Australia; h School of Life Sciences, Kyungpook National University, Daegu, Korea; i Department of Physiology, Juntendo University Graduate School of Medicine, Tokyo, Japan; j Institute for Genetic Medicine, Hokkaido University, Sapporo, Japan

**Keywords:** Autophagy, GABARAP, LIR motif, mammalian ATG8, RavZ protein, selective mATG8–PE delipidation

## Abstract

Mammals conserve multiple mammalian Atg8-family proteins (mATG8s) consisting of GABARAP (GABA type A receptor-associated protein) and MAP1LC3/LC3 (microtubule associated protein 1 light chain 3) subfamilies that tightly bind to autophagic membranes in a membrane-anchored form. These proteins are crucial for selective autophagy and recruit proteins bearing LC3-interacting region (LIR) motifs. However, because limited research tools are available, information on the specific roles of each membrane-anchored mATG8 in selective autophagy is scarce. In this study, we identified LIR motifs specific to the membrane-anchored form of each mATG8 and characterized the residues critical for their selective interaction using cell-based assays and structural analyses. We then used these selective LIR motifs to develop probes and irreversible deconjugases that targeted selective membrane-anchored mATG8s in the autophagic membrane, revealing that membrane-anchored GABARAP subfamily proteins regulate the aggrephagy of amyotrophic lateral sclerosis-linked protein aggregates. Our tools will be useful for elucidating the functional significance of each mATG8 protein on autophagic membranes in autophagy research.

**Abbreviations:**

A:C autophagic membrane:cytosol; ALS amyotrophic lateral sclerosis; ATG4 autophagy related 4; Atg8 autophagy related 8; BafA1 bafilomycin A_1_; BNIP3L/Nix BCL2 interacting protein 3 like; CALCOCO2/NDP52 calcium binding and coiled-coil domain 2; EBSS Earle’s balanced salt solution; GABARAP GABA type A receptor-associated protein; GST glutathione S transferase; HKO hexa knockout; K_d_ dissociation constant; LIR LC3-interacting region; MAP1LC3/LC3 microtubule associated protein 1 light chain 3; NLS nuclear localization signal/sequence; PE phosphatidylethanolamine; SpHfl1 *Schizosaccharomyces pombe*organic solute transmembrane transporter; SQSTM1/p62 SQSTM1/p62; TARDBP/TDP-43 TAR DNA binding protein; TKO triple knockout

## Introduction

Macroautophagy/autophagy is an evolutionarily conserved cellular degradation pathway that selectively or non-selectively eliminates unwanted materials, such as damaged organelles and harmful cytosolic aggregates, to protect the cell’s ability to regulate homeostasis, adapt to various stressors, differentiate during development, and prevent genomic damage [1]. Recent studies on the role of autophagy in secretion and exocytosis have expanded our understanding of its biological significance [2,3]. Furthermore, autophagy dysfunction has been linked to several human diseases including cancer, neurodegenerative diseases, infectious diseases, liver diseases, and cardiovascular disorders [4,5].

Autophagy is tightly regulated by several ATG (autophagy related) proteins. To date, more than 40 *ATG* genes have been identified in yeast and more complex eukaryotes, constituting a diverse family of genes whose products not only precisely control autophagy, but also play roles in membrane trafficking and signaling pathways [1]. ATG8 is a small ubiquitin-like protein in yeast that is covalently conjugated to phosphatidylethanolamine (PE) in phagophores following proteolytic cleavage of its C terminus by the cysteine protease ATG4. ATG4 is also involved in the delipidation of ATG8–PE to release Atg8 from autophagosomes. This cleavage is necessary for the expansion and closure of the phagophore membrane. Although there is only one ATG8 protein in yeast, mammals have two subgroups: the family of MAP1LC3/LC3 (microtubule associated protein 1 light chain 3) proteins (LC3A, LC3B, LC3B2 and LC3C) and GABARAP (GABA type A receptor-associated protein) proteins (GABARAP, GABARAPL1, and GABARAPL2) [6]. These proteins undergo reversible lipidation via conjugation of PE to their C-terminal regions on the phagophore. Although mammalian ATG8 protein (mATG8) conjugation is crucial for conventional autophagy processes, including autophagosome biogenesis and maturation, accumulating evidence indicates its involvement in nonconventional autophagy processes, such as secretory autophagy, LC3-associated phagocytosis, entosis, micropinocytosis, and LC3-associated endocytosis [7]. However, the biological relevance of mATG8 diversity in conventional and non-conventional autophagy or even in autophagy-independent pathways is largely unknown.

mATG8 proteins recruit autophagic machinery containing LC3-interacting region (LIR) motifs into phagophores [8,9]. They can also sequester selective cargo into phagophores via LIR motif-containing receptors during selective autophagy [10]. Therefore, the specific roles of LC3 and GABARAP subfamily proteins are regulated by several LC3- and GABARAP-interacting proteins with LIR motifs. Many mATG8-interacting proteins contain canonical LIR motifs that have a basic hydrophobic LIR motif with a core consensus sequence of (W/F/Y)-X-X-(L/I/V), which binds to the W- and L-sites conserved in mATG8 proteins using the amino acids W/F/Y and L/I/V, respectively [8,11–14]. The GABARAP-interacting motif (GIM) was recently proposed to have a core consensus sequence of (W/F)-(I/V)-X-V, similar to that of a LIR motif [15]. However, some mATG8-interacting proteins contain non-canonical LIR motifs that do not meet the sequence requirements for canonical LIR motifs and present different structural determinants involved in mATG8 interactions [16,17]. Many LIR motifs that interact with mATG8 have been extensively investigated in recent studies [9,18,19]. However, the mechanism underlying selective binding remains largely unknown. Several studies have used peptide-based arrays, glutathione S transferase (GST) affinity-isolation assays, or competitive time-resolved Förster/fluorescence resonance energy transfer to examine the binding properties of LIR motifs to mATG8 [9,18,20–22]. Although these approaches are useful, the assays are purely *in vitro* and may only reflect the binding properties of the LIR motifs of soluble cytosolic mATG8 proteins. Thus, many *in vitro* assay results may not reflect the physiological binding properties of membrane-anchored mATG8 proteins on autophagic membranes in cells. Therefore, identification of functional LIR motifs and characterization of the determinants that result in selective binding of membrane-anchored mATG8 proteins to autophagic membranes in cells is crucial.

In this study, we used novel cellular assays to determine the binding properties of LIR motifs to cytosolic and membrane-anchored mATG8 proteins. We used structural analyses to identify LC3C-, GABARAPL2-, and GABARAP and GABARAPL1-selective LIR motifs and characterized the biochemical properties responsible for their selective binding. Furthermore, we generated a new system that selectively monitors or delipidates mATG8s anchored on the autophagic membrane by incorporating the identified selective LIR motifs into RavZ, an irreversible deconjugase for mATG8. Finally, the use of these selective probes or deconjugases for mATG8–PE revealed that GABARAP subfamily proteins regulate the cellular degradation of amyotrophic lateral sclerosis (ALS)-linked TDP-25 (the 25-kDa C-terminal fragment of TARDBP/TDP-43) protein aggregates during aggrephagy. These data demonstrated that our newly developed tools can be widely applied to elucidate the functional significance of membrane-anchored forms of each mATG8 protein in diverse autophagic and non-autophagic processes.

## Results

### Characterization of binding properties of LIR motifs toward cytosolic and membrane-anchored mATG8s in hexa mATG8-knockout HeLa (HKO) cells

**Figure 1.**
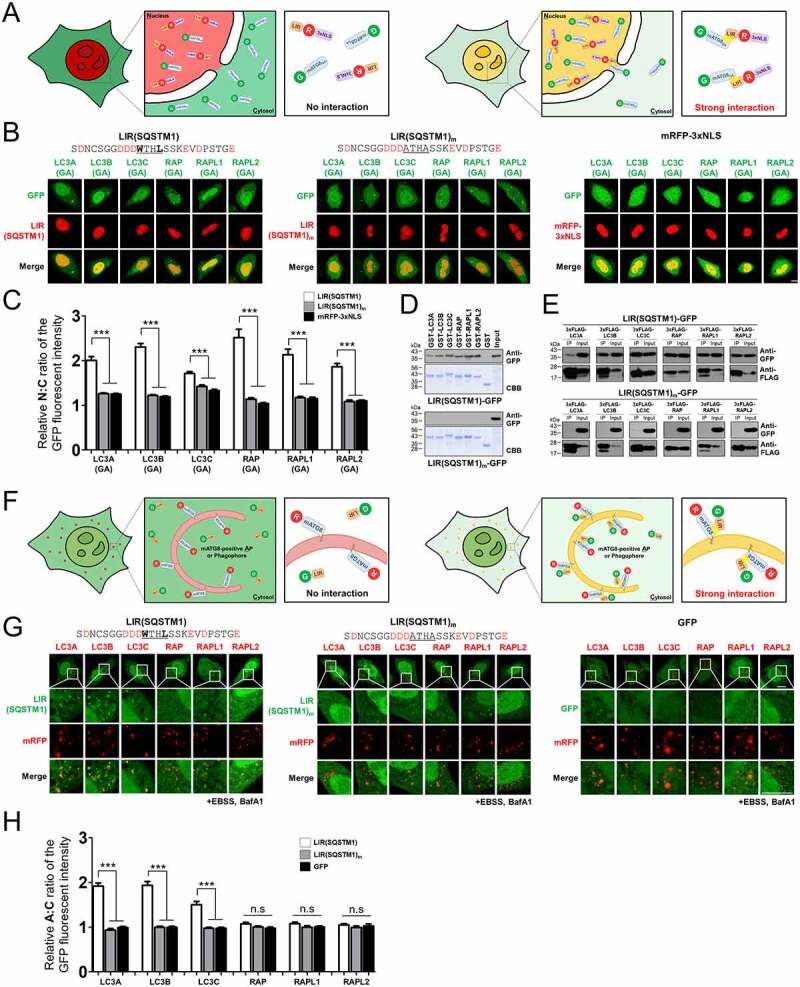
Development of new methods to detect the binding affinities of LIR motifs for cytosolic or membrane-anchored mATG8 proteins in cells. (A) Schematic model for the detection of binding affinities of LIR motifs toward soluble/cytosolic mATG8 proteins in HKO cells using LIR-mRFP-3xNLS and GFP-mATG8(GA) constructs. If a LIR motif interacts with a GFP-tagged mATG8 in the cytosol, the LIR-mRFP-3xNLS should sequester the cytosolic mATG8 in the nucleus (right). If it does not, or weakly interacts with GFP-tagged mATG8(GA), the distribution of GFP-mATG8(GA) will not be changed (left). LIR; LC3-interacting region motif, NLS; nuclear localization signal/sequence, mATG8(GA); mammalian Atg8 mutants in which the C-terminal glycine residue was replaced with alanine to impair PE conjugation (lipidation), LIR-R-3xNLS; LIR(x)-mRFP-3xNLS, G-mATG8_GA_; GFP-mATG8(GA). (B-C) the LIR motif from SQSTM1 (LIR[SQSTM1]) interacts with all cytosolic mATG8 proteins. (B) Cellular localization of LIR(SQSTM1)-mRFP-3xNLS and LIR(SQSTM1)_m_-mRFP-3xNLS, a LIR motif mutant, in which two amino acids in the core sequence of LIR(SQSTM1) were mutated to alanine or mRFP-3xNLS in HKO cells. (C) Quantification of the nuclear and cytosolic (N:C) ratio of GFP fluorescence. *** P < 0.001, one-way ANOVA in conjunction with the Newman – Keuls multiple comparison test. Values are presented as means + SEM (*n* ≥20 for each group). (D-E) Glutathione S transferase (GST) affinity-isolation assays (D) and 3xFlag co-immunoprecipitation (co-IP) experiments (E) to analyze the binding of LIR(SQSTM1)-GFP and LIR(SQSTM1)_m_-GFP. the co-precipitated LIR(SQSTM1)-GFP proteins (upper panel) were analyzed by western blot using the indicated anti-GFP antibodies. The immobilized GST fusion proteins used are displayed on coomassie brilliant blue (CBB)-stained gels (lower panel). Data from one representative experiment of three independent experiments is presented. (F) Schematic model of the detection of binding affinities of LIR motifs toward membrane-anchored mATG8 proteins in HKO cells using LIR(x)-GFP and mRFP-mATG8 constructs. If a LIR motif interacts with an autophagic membrane-anchored mRFP-mATG8, the localization of LIR(X)-GFP will be shifted from the cytosol to the mRFP-mATG8-containing autophagic membrane in cells (right). If the interaction does not occur or is weak, the localization of LIR(X)-GFP will be not changed (left). (G-H) the LIR motif from SQSTM1 (LIR[SQSTM1]) interacts with membrane-anchored LC3 subfamily proteins preferentially. (G) Cellular localization of LIR(SQSTM1)-, LIR(SQSTM1)_m_-GFP, or GFP and each mRFP-mATG8 in HKO cells upon autophagy induction (100 nM bafilomycin A_1_ (BafA1) in Earle’s balanced salts solution (EBSS) for 2 h, +EBSS, BafA1). Scale bar: 10 μm. (H) Quantification of the autophagosome and cytosol (A:C) ratio of GFP fluorescence. *** P < 0.001; n.S. not significant, one-way ANOVA in conjunction with the Newman – Keuls multiple comparison test. Values are presented as means + SEM (*n* ≥20 for each group). Scale bar: 10 μm. X(GA), GFP-X(GA); X, mRFP-X (X = LC3A, LC3B, LC3C, GABARAP, GABARAPL1, or GABARAPL2); LIR(SQSTM1), LIR(SQSTM1)-mRFP-3xNLS or LIR(SQSTM1)-GFP; LIR(SQSTM1)_m_, LIR(SQSTM1)_m_-mRFP-3xNLS or LIR(SQSTM1)_m_-GFP.

To examine the binding properties of LIR motifs fo each cytosolic mATG8 and to identify selective mATG8-binding LIR motifs in cells, we first used a LIR motif tagged with a monomeric red fluorescent protein (mRFP) fused to a 3xNLS sequence (LIR-mRFP-3xNLS) for a nuclear localization signal or sequence (NLS) assay in HKO cells, in which endogenous mATG8s were not expressed as described previously [23] (Figure S1; Figure 1A). If a LIR motif interacts with a green fluorescent protein (GFP)-tagged mATG8 in the cytosol, the LIR-mRFP-3xNLS sequesters cytosolic mATG8 in the nucleus, depending on the binding preference of the LIR-mRFP-3xNLS protein in HKO cells (Figure 1A). This enabled the quantification of the relative GFP fluorescence intensity ratio between the nucleus and cytosol (N:C ratio) in live cells. To test this, we used GFP-tagged mATG8 mutants (GFP-mATG8[GA]), in which the C-terminal glycine residue was replaced with alanine to impair PE conjugation (lipidation), leading to inhibition of cellular localization to phagophores and consequently autophagosomes. We first examined the binding properties of a LIR motif from SQSTM1/p62 (LIR[SQSTM1]), the first LIR motif for mATG8 proteins identified in the autophagic membrane [24]. Each GFP-mATG8(GA) protein was primarily localized to the nucleus in cells expressing LIR(SQSTM1)-mRFP-3xNLS but not in cells expressing LIR motif mutant LIR(SQSTM1)_m_-mRFP-3xNLS, a LIR motif mutant, in which two amino acids in the core sequence of LIR(SQSTM1) were mutated to alanine or mRFP-3xNLS (Figure 1B). The relative N:C ratio in LIR(SQSTM1)-mRFP-3xNLS-expressing cells was significantly higher than that in LIR(SQSTM1)_m_-mRFP-3xNLS- or mRFP-3xNLS-expressing cells for all mATG8s, indicating that this LIR bound to all types of cytosolic mATG8 in cells in a LIR-dependent manner (Figures 1B,C). Consistent with the N:C ratio assay, LIR(SQSTM1)-GFP, but not LIR(SQSTM1)_m_-GFP, bound to all mATG8 proteins in GST pull-down and co-immunoprecipitation (co-IP) experiments using GST-mATG8 or 3xFlag-mATG8 proteins, respectively (Figures 1D,E), confirming that LIR(SQSTM1) bound to all cytosolic mATG8 proteins.

Next, we determined the binding properties of LIR motifs to autophagic membrane-anchored mATG8s. Thus, the translocation of the LIR motif from the cytosol to the mRFP-mATG8-containing autophagic membrane during their interaction was observed (Figure 1F). Therefore, if the ratio of GFP fluorescence intensity between the autophagic membrane and cytosol (A:C ratio) is compared, the relative binding affinity of a LIR motif for each expressed membrane-anchored mRFP-mATG8 can be determined (Figure 1F). To maximize visual autophagosomes in cells overexpressing each mRFP-mATG8(GA) and either LIR(SQSTM1)-GFP, LIR(SQSTM1)_m_-GFP, or GFP, we treated HKO cells with 100 nM bafilomycin A_1_ (BafA1) blocking for autophagosome-lysosome fusion under starvation induction (Earle’s balanced salt solution [EBSS]) for 2 h. As shown in Figure S2, an mRFP-tagged LC3B protein exclusively localized to autophagosomes, whereas each mRFP-mATG8(GA) protein diffusely localized mainly to the cytosol and nucleus in HKO cells treated with 100 nM BafA1 under starvation induction for 2 h, indicating that unlipidated mATG8s are expressed diffusely in cells. To validate the binding of the SQSTM1 LIR motif to mATG8 proteins anchored to the autophagic membrane, we expressed LIR(SQSTM1)-GFP, LIR(SQSTM1)_m_-GFP, or GFP with each mRFP-mATG8 in HKO cells treated with 100 nM BafA1 in EBSS for 2 h. We then quantified the relative GFP fluorescence intensity ratio between the autophagosome and cytosol (A:C ratio) (Figures 1G,H). As expected, the relative A:C ratio in LIR(SQSTM1)-GFP-expressing cells was significantly higher than that in LIR(SQSTM1)_m_-GFP- or GFP-expressing cells for the LC3 subfamily. Unexpectedly, the relative A:C ratio in LIR(SQSTM1)-GFP-expressing cells was not significantly different from that in LIR(SQSTM1)_m_-GFP- or GFP-expressing cells for the GABARAP subfamily (Figures 1G,H). Our results indicated that LIR(SQSTM1)-GFP preferentially localized to membrane-anchored LC3 subfamily proteins, but not to membrane-anchored GABARAP subfamily proteins in cells. Taken together, our data raise the possibility that LIR(SQSTM1) could interact with all types of cytosolic mATG8s but was targeted to the autophagic membrane in cells, probably via preferential binding to LC3 subfamily proteins anchored to the autophagic membrane in cells. Consistent with this, it has been reported that SQSTM1 binds to both soluble LC3B and GABARAPL2 in the cytosol but only to autophagic membrane-anchored LC3B [25]. Therefore, our newly developed methods can identify the binding properties of LIR motifs for cytosolic or membrane-anchored mATG8s in cells, respectively.

### Identification of LIR motifs that bind to autophagic membrane-anchored mATG8

**Figure 2.**
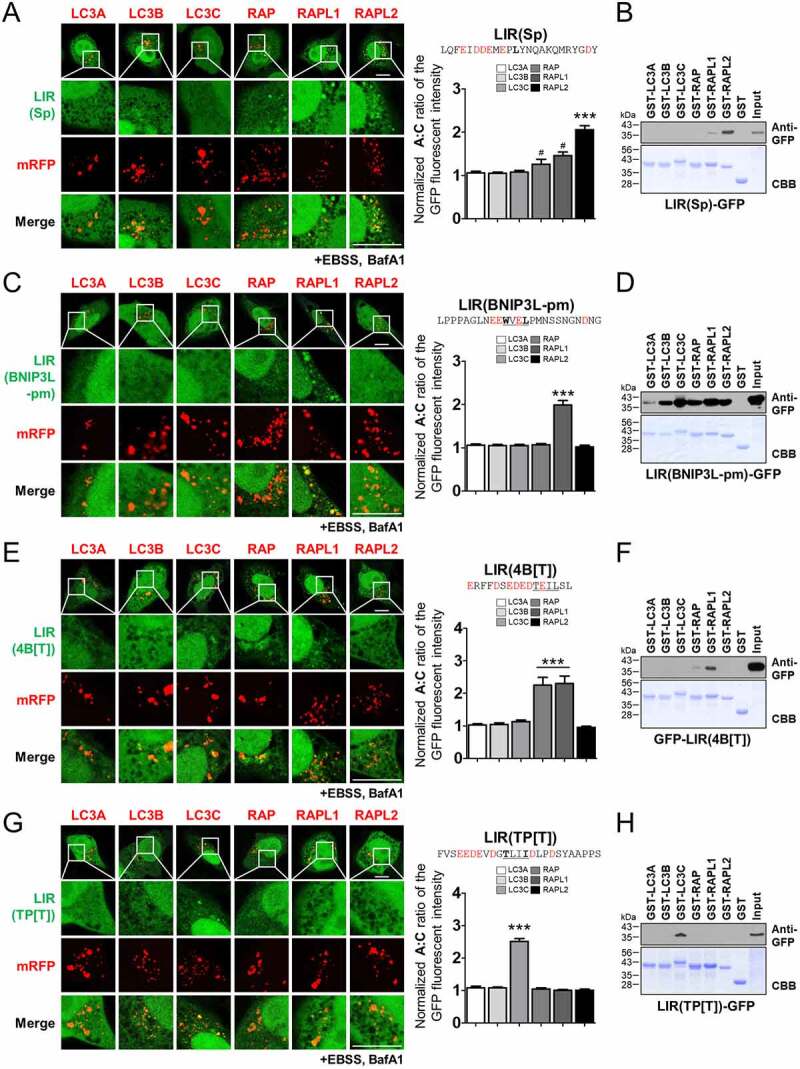
Identification of selective mATG8-binding LIR motifs. The LC3-interacting region (LIR) from SpHfl1 (LIR[Sp]) (A-B), a phosphomimetic LIR motif from BNIP3L/Nix (LIR(BNIP3L-pm) (C-D), a LIR motif from the ATG4B mutant LIR(4B[T]) (E-F), and a LIR motif from the TP53INP2 mutant LIR(TP[T]) (G-H) interact preferentially with GABARAPL2, GABARAPL1, GABARAP and GABARAPL1, and LC3C, respectively. (A, C, E, G) Cellular localization of each mRFP-mATG8 with LIR(Sp)-GFP (A), LIR(BNIP3L-pm)-GFP (C), LIR(4B[T]) -GFP (E), or LIR(TP[T])-GFP (G) in HKO cells upon autophagy induction (100 nM bafilomycin A_1_ [BafA1]) in Earle’s balanced salt solution (EBSS) for 2 h (+EBSS, BafA1) (left) and quantification of the autophagosome and cytosol (A:C) ratio of GFP fluorescence (right). The A:C ratio was normalized to that of GFP and each mRFP-mATG8-expressing cell, as shown in Figures 1G,H (the normalized A:C ratio). Values are presented as the mean ± SEM (*n* ≥20 for each group). ***P < 0.001 compared with all other mRFP-mATG8 expressing groups with one-way ANOVA in conjunction with the Newman – Keuls multiple comparison test. # P < 0.05, compared with GFP + the corresponding mATG8-expressing cells shown in Figure 1H using a two-tailed student’s *t*-test. (B, D, F, G) Glutathione S transferase (GST) pull-down assays to analyze the binding of LIR(Sp)-GFP (B), LIR(BNIP3L-pm)-GFP (D), LIR(4B[T])-GFP (F), and LIR(TP[T])-GFP (H). The co-precipitated LIR-GFP protein was analyzed by western blotting using the indicated anti-GFP antibodies. X, mRFP-X (X= LC3A, LC3B, LC3C, GABARAP, GABARAPL1, or GABARAPL2). For the GST pull-down assay, data from one representative experiment of three independent experiments are presented. Scale bar: 10 μm.

As our goal was to generate a new system that selectively monitors or delipidates autophagic membrane-anchored mATG8s, the A:C ratio assay was a useful strategy for identifying LIR motifs that selectively bind to autophagic membrane-anchored mATG8s. Therefore, we examined the binding properties of various GFP-fused, known LIR motifs with mRFP-mATG8 proteins in HKO cells using the A:C ratio assay (Table 1; Figure S3; Figure 2). To this end, we expressed each LIR(X)-GFP (X: the protein from which the LIR motif originated) together with each mRFP-mATG8 in HKO cells treated with 100 nM BafA1 in EBSS for 2 h. We then quantified the A:C ratio of each mATG8. Finally, the value was normalized to that of GFP and each mRFP-mATG8-expressing cell, as shown in Figures 1G,H (the normalized A:C ratio).

**Table 1.** Summary of the normalized binding property of known LIR motifs to each membrane-anchored mATG8 in HKO cells treated with 100 nM BafA1 in EBSS for 2 h.

LIR protein	LIR motif	LC3/GABARAP family interaction					
		LC3A	LC3B	LC3C	GABARAP	GABARAPL1	GABARAPL2
SpHfl1(I) (Sp(I))	LQFEIDDEMEPIYNQAKQMRYGDY	-	-	-	-	-	**++**
		(1.059 ± 0.033, N = 15)	(1.05 ± 0.04, N = 15)	(0.9478 ± 0.048, N = 16)	(1.013 ± 0.053, N = 15)	(0.8723 ± 0.039, N = 16)	(2.549 ± 0.166, N = 15)
WDFY3/ALFY	DQLSLDEKDG**F**IF**V**NYSEGQTRAHL	-	-	**+**	**++**	**++**	**++**
		(1.345 ± 0.041, N = 15)	(1.287 ± 0.065, N = 15)	(1.763 ± 0.09, N = 15)	(3.236 ± 0.237, N = 15)	(3.064 ± 0.187, N = 15)	(3.118 ± 0.276, N = 15)
AS_67 peptide	SFTMYEPDQQ**T**IV**I**ES	-	-	**++**	-	-	-
		(1.154 ± 0.045, N = 20)	(1.153 ± 0.046, N = 21)	(2.532 ± 0.125, N = 20)	(1.042 ± 0.045, N = 20)	(1.053 ± 0.035, N = 25)	(0.9901 ± 0.047, N = 20)
ATG4A	QLEEFDLEED**F**EI**L**SV	**++**	**++**	-	**++**	**++**	**+**
		(2.695 ± 0.1, N = 32)	(2.95 ± 0.119, N = 30)	(1.316 ± 0.042, N = 34)	(2.174 ± 0.133, N = 27)	(2.181 ± 0.118, N = 30)	(1.415 ± 0.07, N = 30)
ATG4B (4B)	ERFFDSEDED**F**EI**L**SL	**++**	**++**	-	**++**	**++**	**++**
		(2.707 ± 0.118, N = 32)	(2.707 ± 0.108, N = 31)	(1.248 ± 0.069, N = 30)	(2.299 ± 0.108, N = 30)	(2.189 ± 0.122, N = 30)	(2.084 ± 0.139, N = 30)
ATG4C	KQLKRFSTEE**F**VL**L**	**++**	**++**	**+**	**+**	**++**	**+**
		(2.177 ± 0.079, N = 34)	(2.14 ± 0.088, N = 32)	(1.487 ± 0.055, N = 30)	(1.884 ± 0.094, N = 22)	(2.927 ± 0.122, N = 29)	(1.869 ± 0.092, N = 28)
ATG4D	LRAKRPSSED**F**VF**L**	**++**	**++**	**+**	**+**	**++**	**+**
		(2.204 ± 0.123, N = 30)	(2.209 ± 0.104, N = 30)	(1.597 ± 0.067, N = 30)	(1.825 ± 0.069, N = 30)	(2.653 ± 0.113, N = 30)	(1.952 ± 0.088, N = 30)
ATG13	GGSSGNTHDD**F**VM**I**DFKPAFSKDDI	-	-	-	-	-	-
		(1.284 ± 0.071, N = 16)	(1.225 ± 0.092, N = 18)	(1.2 ± 0.069, N = 15)	(1.171 ± 0.05, N = 18)	(1.081 ± 0.051, N = 17)	(1.07 ± 0.051, N = 15)
BNIP3	GMQEESLQGS**W**VE**L**HFSNNGNGGSV	**+**	**+**	**+**	**+**	**+**	**+**
		(1.61 ± 0.12, N = 15)	(1.678 ± 0.114, N = 15)	(1.588 ± 0.133, N = 17)	(1.615 ± 0.116, N = 15)	(1.452 ± 0.063, N = 15)	(1.532 ± 0.08, N = 15)
FAIM2	APTAVPLHPS**W**AY**V**DPSSSSSYDNG	**+**	**+**	**+**	-	-	-
		(1.952 ± 0.103, N = 21)	(1.996 ± 0.1, N = 22)	(1.845 ± 0.106, N = 20)	(1.371 ± 0.05, N = 22)	(1.257 ± 0.05, N = 21)	(1.313 ± 0.04, N = 20)
RETREG1/FAM134B	EDTDTEEGDD**F**EL**L**DQSELDQIESE	**++**	**+**	-	**+**	**++**	-
		(2.097 ± 0.101, N = 15)	(1.815 ± 0.089, N = 15)	(0.9764 ± 0.054, N = 16)	(1.874 ± 0.163, N = 15)	(2.147 ± 0.135, N = 15)	(1.009 ± 0.034, N = 18)
RB1CC1/FIP200	PDSIDAHTFD**F**ET**I**PHPNIEQTIHQ	-	-	-	-	-	-
		(1.208 ± 0.042, N = 20)	(1.182 ± 0.077, N = 21)	(1.207 ± 0.056, N = 21)	(1.175 ± 0.057, N = 21)	(1.135 ± 0.033, N = 20)	(1.108 ± 0.043, N = 24)
FUNDC1	PQDYESDDDS**Y**EV**L**DLTEYARRHQW	**+**	**++**	**++**	**+**	**+**	**+**
		(1.9 ± 0.11, N = 16)	(2.087 ± 0.104, N = 15)	(2.305 ± 0.15, N = 15)	(1.953 ± 0.137, N = 17)	(1.955 ± 0.154, N = 17)	(1.777 ± 0.123, N = 15)
FYCO1 (Fy)	RPPDDAV**F**DI**I**TDEELCQIQESGS	**++**	**+++**	**+**	-	-	-
		(3.853 ± 0.151, N = 20)	(4.054 ± 0.139, N = 20)	(1.886 ± 0.099, N = 20)	(1.225 ± 0.055, N = 20)	(1.124 ± 0.06, N = 20)	(1.139 ± 0.033, N = 20)
JMY	FALEETLESD**W**VA**V**RPHVFDEREKH	-	-	-	**+**	**+**	**+**
		(1.273 ± 0.084, N = 15)	(1.217 ± 0.086, N = 16)	(1.304 ± 0.081, N = 16)	(1.465 ± 0.08, N = 17)	(1.459 ± 0.141, N = 15)	(1.415 ± 0.115, N = 15)
NBR1	QSQSSASSED**Y**II**I**LPECFDTSRPL	-	-	-	-	-	-
		(1.12 ± 0.035, N = 16)	(1.099 ± 0.042, N = 15)	(1.137 ± 0.039, N = 18)	(1.01 ± 0.043, N = 15)	(1.027 ± 0.049, N = 15)	(1.017 ± 0.045, N = 15)
BNIP3L/Nix	LPPPAGLNSS**W**VE**L**PMNSSNGNDNG	-	-	-	-	-	-
		(0.9214 ± 0.028, N = 15)	(1.046 ± 0.042, N = 15)	(1 ± 0.036, N = 16)	(1.015 ± 0.045, N = 15)	(1.067 ± 0.033, N = 15)	(1.002 ± 0.037, N = 15)
PLEKHM1	QKVRPQQEDE**W**VN**V**QYPDQPEEPPE	**+**	-	**+**	**++**	**+**	**+**
		(1.451 ± 0.096, N = 15)	(1.29 ± 0.067, N = 15)	(1.574 ± 0.113, N = 15)	(2.115 ± 0.133, N = 15)	(1.861 ± 0.095, N = 15)	(1.894 ± 0.127, N = 15)
STBD1 (St)	NSQDRVDHEE**W**EM**V**PRHSSWGDVGV	-	-	-	**++**	**+++**	**+++**
		(1.026 ± 0.039, N = 20)	(0.9884 ± 0.03, N = 20)	(1.083 ± 0.049, N = 20)	(3.796 ± 0.109, N = 20)	(5.085 ± 0.173, N = 20)	(4.698 ± 0.199, N = 20)
TAX1BP1	TMEDEGNSDMLV**V**TTKAGLLELKIE	-	-	-	-	-	-
		(1.223 ± 0.027, N = 15)	(1.214 ± 0.04, N = 18)	(1.324 ± 0.057, N = 15)	(1.281 ± 0.068, N = 15)	(1.139 ± 0.07, N = 16)	(1.255 ± 0.063, N = 15)
TBC1D25	PSEDSPLLED**W**DI**I**SPKDVIGSDVL	**++**	**++**	**+**	-	**+**	-
		(2.356 ± 0.133, N = 21)	(2.3 ± 0.122, N = 22)	(1.647 ± 0.057, N = 25)	(1.36 ± 0.052, N = 23)	(1.408 ± 0.107, N = 23)	(1.278 ± 0.047, N = 22)
TP53INP1	PEFNEKEDDE**W**IL**V**DFIDTCTGFSA	**++**	**+**	**++**	**++**	**++**	**++**
		(2.04 ± 0.099, N = 15)	(1.819 ± 0.092, N = 15)	(2.895 ± 0.137, N = 15)	(2.309 ± 0.173, N = 15)	(2.434 ± 0.191, N = 15)	(3.141 ± 0.225, N = 15)
TP53INP2 (TP)	FVSEEDEVDG**W**LI**I**DLPDSYAAPPS	**++**	**++**	**+++**	**+++**	**+++**	**+++**
		(2.538 ± 0.051, N = 27)	(2.464 ± 0.096, N = 20)	(4.702 ± 0.159, N = 20)	(5.408 ± 0.141, N = 20)	(5.003 ± 0.141, N = 20)	(4.201 ± 0.17, N = 20)
UBR4	QEQSEVDHGD**F**EM**V**SESMVLETAEN	**+**	**+**	**+**	**++**	**++**	**+**
		(1.5 ± 0.098, N = 15)	(1.541 ± 0.105, N = 15)	(1.773 ± 0.2, N = 15)	(2.315 ± 0.142, N = 15)	(2.811 ± 0.16, N = 15)	(1.524 ± 0.101, N = 16)
ULK1	SKDSSCDTDD**F**VM**V**PAQFPGDLVAE	-	-	-	**+**	**+**	**+**
		(1.057 ± 0.033, N = 28)	(1.145 ± 0.027, N = 28)	(1.263 ± 0.037, N = 23)	(1.649 ± 0.077, N = 23)	(1.584 ± 0.073, N = 24)	(1.582 ± 0.089, N = 26)
ULK2	SKNSSCDTDD**F**VL**V**PHNISSDHSCD	-	-	**+**	**++**	**++**	**+**
		(1.023 ± 0.038, N = 15)	(0.9548 ± 0.042, N = 15)	(1.427 ± 0.073, N = 15)	(2.386 ± 0.182, N = 15)	(2.497 ± 0.158, N = 15)	(1.911 ± 0.154, N = 17)

Underline; core region in LIR motif. The normalized A:C ratio: −1.4: -; 1.4–2.0: +; 2.0–4.0: ++; 4.0: +++

AS_67 is a LC3C-specific peptide originated from a previous report [15].

We first identified LIR motifs that preferentially bind to GABARAPL2 (Figures 2A,B). An atypical LIR motif from SpHfl1 (LIR[Sp]), which binds to Atg8 in yeast [26], was mostly localized to GABARAPL2- and weakly but significantly localized to GABARAP- and GABARAPL1-containing autophagic membranes (Figure 2A). Intriguingly, LIR(Sp)-GFP exhibited strong binding to cytosolic GABARAPL2 and weak binding to GABARAPL1 in a GST pull-down assay (Figure 2B). Isothermal titration calorimetry (ITC) experiments further confirmed the strong and specific binding affinity of LIR(Sp) to GABARAPL2; the dissociation constant (K_d_) of LIR(Sp) to GABARAPL2 was 0.24 μM, which was 8–12 times lower than that to GABARAP (1.8 μM) and GABARAPL1 (2.9 μM) (Figure S4A). The corresponding values for the LC3 subfamily proteins could not be determined because the interactions were too weak (Figure S4A). Thus, LIR(Sp) preferentially binds cytosolic and membrane-anchored GABARAPL2.

Next, we identified the LIR motif that preferentially localized to GABARAPL1-containing autophagic membranes (Figures 2C,D). The LIR motif from BNIP3L/Nix (LIR[BNIP3L]) exhibited no significant binding to any mATG8 (Table 1 and Figure S3). However, a phosphomimetic mutant of BNIP3L (LIR[BNIP3L-pm]), in which two serine residues located at the N terminus of the core LIR motif were substituted by phosphomimetic E residues, exhibited selective localization to GABARAPL1-containing autophagic membranes [27] (Figure 2C). In contrast, in the GST affinity-isolation assay, LIR(BNIP3L-pm) interacted with all the mATG8 proteins (Figure 2D). Intriguingly, these results suggest the possibility that LIR(BNIP3L-pm) binds to all cytosolic mATG8 proteins but selectively binds to autophagic membrane-anchored GABARAPL1 in cells.

Additionally, we identified the LIR motif that preferentially localizes to GABARAP- and GABARAPL1-containing autophagic membranes (Figures 2E,F). The LIR motif from the C terminus of ATG4B (LIR[4B]) is bound to all autophagic membrane-anchored mATG8 proteins, except for LC3C (Table 1 and Figure S3). A previous two-dimensional peptide array scan showed that the replacement of F with S/T within the core region of LIR(4B) severely impaired binding to LC3B, but not to GABARAP [28]. Consistent with this report, a mutation of F to T within the core region of LIR(4B), referred to as LIR(4B[T]), which makes this motif atypical, almost diminished binding to the LC3 subfamily and GABARAPL2, but retained significant binding to GABARAP and GABARAPL1 (Figures 2E,F). Thus, LIR(4B[T]) selectively binds to cytosolic and membrane-anchored GABARAP and GABARAPL1.

Finally, we identified a LIR motif that preferentially localized to LC3C-containing autophagic membranes (Figures 2G,H). GFP fused to the LIR motif of TP53INP2 (LIR[TP]) bound to all membrane-anchored mATG8 proteins (Table 1). Meanwhile, the W to T mutation within the core region of LIR(TP), referred to as LIR(TP[T]), which makes this LIR motif atypical, induced selective binding to membrane-anchored LC3C (Figure 2G). Our GST pull-down assay confirmed the selective binding of LIR(TP[T])-GFP to LC3C (Figure 2H). ITC experiments also demonstrated the selectivity of LIR(TP[T]) toward LC3C (Figure S4B). The K_d_ value of LIR(TP[T]) binding to LC3C was 26.9 μM, five times lower than that of the other mATG8 proteins (>132 μM), indicating that LIR(TP[T]) could be used as an LC3C-selective LIR motif (Figure S4B). Taken together, we successfully identified selective LIR motifs for membrane-anchored forms of LC3C, GABARAP and GABARAPL1, GABARAPL1, and GABARAPL2.

### Structural basis for selective binding of mATG8-binding LIR motifs

**Figure 3.**
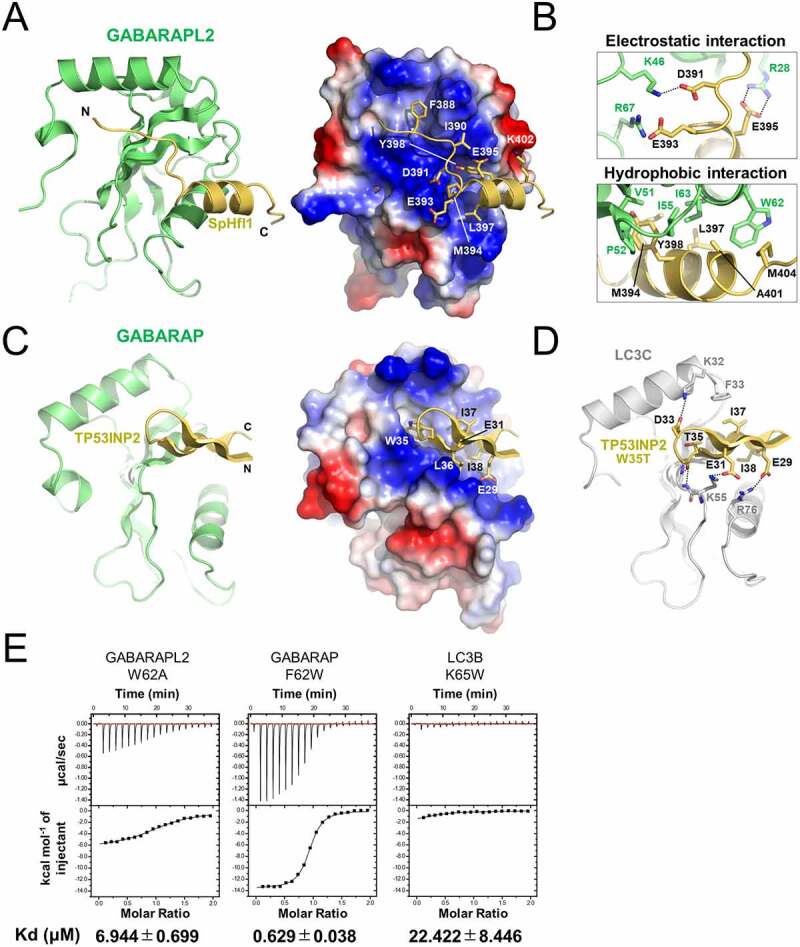
Structural basis for selective binding of mATG8-binding LIR motifs. (A, C) Crystal structure of SpHfl1 LIR-GABARAPL2 fusion (A) and LIR(TP)-GABARAP fusion (C), both models of which were prepared by combining GABARAP and GABARAPL2 from one fusion molecule and LIR from another fusion molecule that interacts with each other in the crystal. Left, ribbon model. Right, electrostatic surface potentials of mATG8 proteins with a ribbon model of LIR peptides, where the side chains of LIR residues involved in the interaction are shown as a stick model. (B) Close-up view of the interactions between SpHfl1 LIR and GABARAPL2. Possible electrostatic interactions are shown as a broken line. (D) Modeled structure of the LIR(TP[T])-LC3C complex. Possible electrostatic or hydrogen-bond interactions are shown with a stick model. (E) ITC data of mATG8 mutants with SpHfl1 LIR.

We attempted to determine the crystal structures of atypical LIRs bound to cytosolic mATG8 proteins to reveal the specific mechanism underlying the interaction between LIR and mATG8 proteins at the atomic level. We successfully determined the structure of the LIR(Sp)-GABARAPL2 fusion protein at a resolution of 1.86 Å (Figure 3A; Table S1). The structure of the LIR(Sp)-GABARAPL2 complex is similar to that of the LIR(Sp)-SpAtg8 complex (Figure S5A). The SpHfl1 LIR consists of an α-helix, from D391 to M404, and an N-terminal tail. The helix formed extensive hydrophobic interactions with V51, P52, I55, W62, and I63 of GABARAPL2 using M394, L397, Y398, A401, and M404. Among these, Y398 formed the most critical interaction, as it was inserted deep into the L-site pocket of GABARAPL2. In addition to hydrophobic interactions, three acidic residues of LIR(Sp), D391, E393, and E395, formed electrostatic interactions with K46, R67, and R28 in GABARAPL2, respectively (Figure 3B) [26]. Although most of the interactions were similar to those in the LIR(Sp)-SpATG8 structure, F388 of SpHfl1 was not inserted into the W-site of GABARAPL2. Considering that the alanine substitution of F388 induced a limited decrease in the binding affinity to SpATG8, this observation implies that W-site binding is not important for the binding of SpHfl1 to Atg8-family proteins. We also performed sequence alignment of mATG8 proteins (Figure S5B) [26]. Among the GABARAPL2 residues involved in the interaction with LIR(Sp), W62 was the sole residue that was not conserved in other mATG8 proteins (F, K, or S was also observed at this position). To assess the importance of W62 in this interaction, we performed ITC experiments using three mATG8 mutants (GABARAPL2 W62A, GABARAP F62W, and LC3B K65W) (Figure 3E). The W62A mutation in GABARAPL2 reduced its binding affinity to LIR(Sp), but the F62W mutation in GABARAP had the opposite effect. K65W mutation in LC3B marginally increased this interaction. Coupled with the fact that SpAtg8, which binds strongly to SpHfl1, has a Y at position 62, these data suggest that either W or Y at position 62 is necessary, but not sufficient, for the strong binding of ATG8 family proteins to SpHfl1 LIR.

The crystal structure of the LIR(TP)-mATG8 complex has not been reported previously. We failed to crystallize the LIR(TP) mutants bound to mATG8 proteins but succeeded in crystallizing and determining the structure of the wild-type (WT) LIR(TP)-GABARAP fusion protein at a resolution of 1.75 Å (Figure 3C; Table S1). The conformation of LIR(TP) was unique compared to that of canonical LIRs, where W35 and I38 bound to the W-site and L-site, respectively, in a canonical manner; the region *N*-terminal to the core LIR sequence (residues 28–33) formed an intramolecular β-sheet with the core LIR sequence (Figure 3C, left). An intramolecular but distinct β-sheet was also observed for the RavZ LIR, with the region C-terminal to the core LIR sequence forming a β-sheet with the core LIR sequence [29]. Although the LIR(TP) peptide used for crystallization possesses six acidic residues, only one residue (E29) forms an electrostatic interaction with GABARAP (R67), suggesting that the binding affinity is largely dependent on the core LIR motif. To assess the specificity of LIR(TP[T]) to LC3C, we prepared a structural model of the LIR(TP[T])-LC3C complex by superimposing the WT LIR(TP)-GABARAP structure onto the LC3C-CALCOCO2/NDP52 structure (Protein Database ID [PDBID]:3VVW) followed by manual model adjustment (Figure 3D) [16]. The CALCOCO2 LIR possessed a non-canonical core sequence (I^133^-L-V-V^136^) and showed specific interaction with LC3C, with V136 binding to the L-site, whereas I133 did not bind to the W-site. The lack of canonical interactions must be compensated by additional interactions to maintain a high binding affinity, including hydrophobic interactions between V135 and LC3C F33 and a hydrogen bond between N129 and LC3C K32. In the case of LIR(TP[T]), I37 and D33 formed hydrophobic and electrostatic interactions with LC3C F33 and K32, respectively. Additionally, E29 and E31 of LIR(TP[T]) form electrostatic interactions with R76 and K55 of LC3C, respectively. Among these residues, K55 and R76 were strictly conserved in all mATG8 proteins, K32 was conserved within the GABARAP subfamily, and F33 was unique to LC3C. These observations suggested that F33 of LC3C was responsible for its observed specificity. Consistent with this, the mutation of K32/F33 to Q/H (corresponding to LC3A or LC3B), but not Y (corresponding to the GABARAP subfamily), reduced the binding affinity of LC3C to LIR(TP[T]) (Figure S6A). We noticed that the α2 helix of the LC3 family, whose C terminus contained K32 and F33, was located closer to the LIR-binding pocket than that of GABARAP subfamily proteins (Figure S5C, left), a feature that seemed to enable K32 and F33 of LC3C to interact with D33 and I37 of LIR(TP[T]) (Figure 3D). The distinct positioning of the α2 helix could be attributed to the amino acid at position 18 (using GABARAP numbering). The LC3 family possesses a V at this position, which has a larger side chain than G (GABARAP and GABARAPL1) and S (GABARAPL2), resulting in the steric crush of the α2 helix with the ubiquitin fold, thereby positioning the α2 helix toward the LIR-binding site (Figure S5C, right). Consistent with this, the mutation of V26 to G (corresponding to GABARAP and GABARAPL1) reduced the association between the LC3C-positive autophagic membrane and LIR(TP[T]) (Figure S6A). Therefore, the combination of K32, F33, and the properly positioned α2 helix is likely to be necessary for the specific binding of LC3C to LIR(TP[T]).

### Development of probes that selectively monitor membrane-anchored mATG8 proteins on autophagosomes

**Figure 4.**
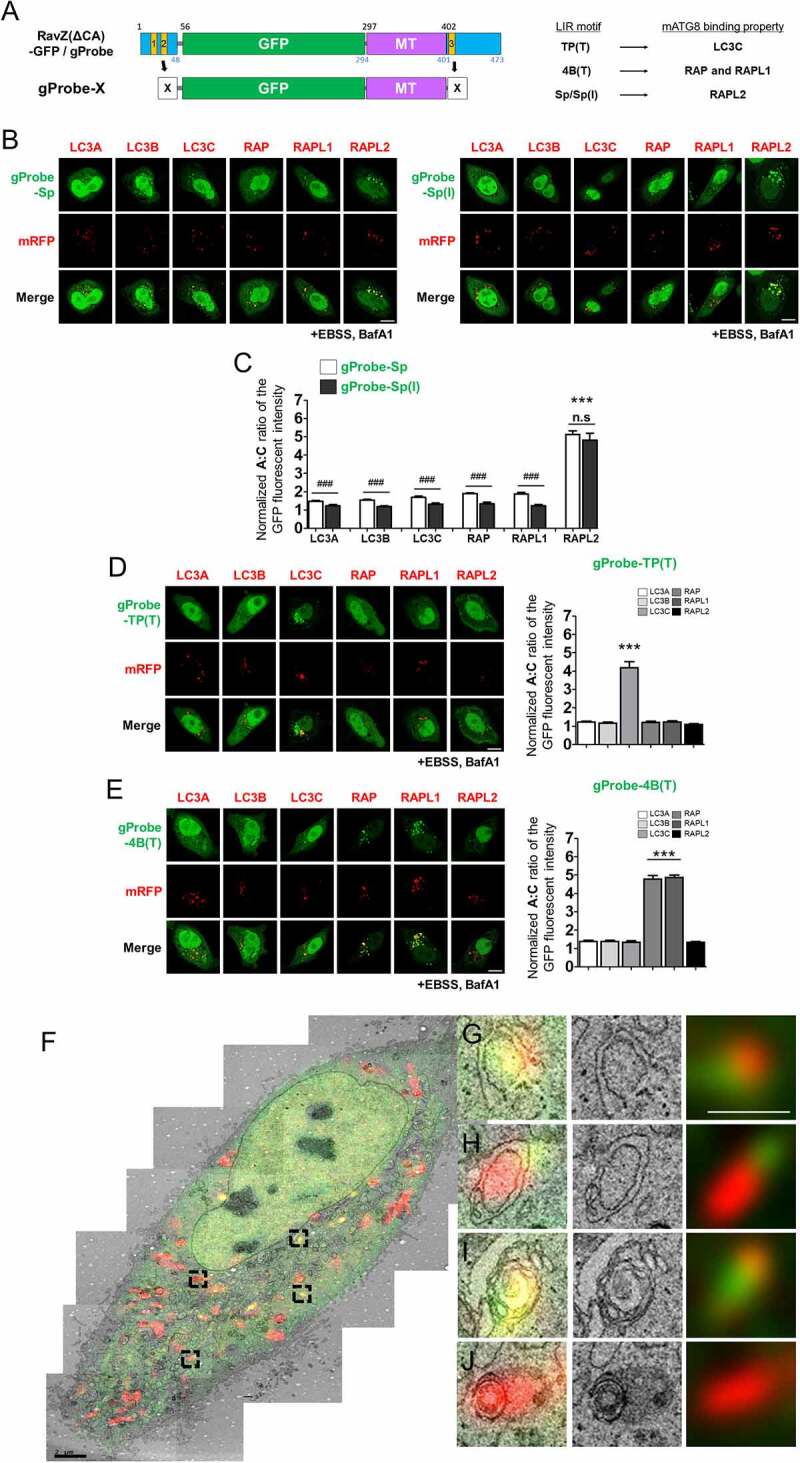
Selective mATG8-containing autophagosome targeting of gProbe-X. A) Schematic diagram of gProbe-X and its binding preference. X: Sp, Sp(I), TP(T), or 4B(T). (B) Cellular localization of the gProbe-X (SP or Sp[I]) and each mRFP-mATG8 in HKO cells upon autophagy induction (100 nM bafilomycin A_1_ [BafA1]) in Earle’s balanced salts solution (EBSS) for 2 h, (+EBSS, BafA1). (C) Quantification of the autophagosome and cytosol (A:C) ratio of GFP fluorescence. The A:C ratio was normalized to that of GFP and each mRFP-mATG8-expressing cells, as shown in Figures 1G,H (the normalized A:C ratio). Values are presented as means + SEM (*n* ≥20 for each group). ***P < 0.001 compared with all other mRFP-mATG8 expressing groups with one-way ANOVA in conjunction with the Newman – Keuls multiple comparison test. ### P < 0.001; n.s, not significant with two-tailed student *t*-test. (D and E) Cellular localization of the gProbe-X (TP[T] and 4B[T]) and each mRFP-mATG8 in HKO cells (left) upon autophagy induction (100 nM bafilomycin A_1_ [BafA1]) in Earle’s balanced salts solution (EBSS) for 2 h, (+EBSS, BafA1) and quantification of the A:C ratio of GFP fluorescence (right). Values are presented as means + SEM (*n* ≥20 for each group). ***P < 0.001 compared with all other mRFP-mATG8 expressing groups with one-way ANOVA in conjunction with the Newman – Keuls multiple comparison test. (F) Correlative light and electron microscopy (CLEM) images of rProbe-Fy and gProbe-Sp(I)-co-expressing HeLa cells. The cells were treated with rapamycin (100 nM) in the presence of BafA1 (100 nM) for 2 h. Red, signifying rProbe-Fy, indicates endogenous membrane-anchored LC3A/B on the autophagic membrane, and green, signifying gProbe-Sp(I), indicates endogenous membrane-anchored GABARAPL2 on the autophagic membrane. Multiple transmission electron microscopy images were taken at 2500× magnification. The images were stitched to create a large field of view. The black dotted-line box shows the morphology of the autophagosome. N, nucleus; M, mitochondria; ER, endoplasmic reticulum. (G-J) Magnified autophagosome image by CLEM. (G) Phagophore, (H) early autophagosome, and (I, J) late autophagosome are shown (left: CLEM, middle: electron microscopy image, right: fluorescence image). Scale bar: 1 μm.

Next, we monitored membrane-anchored mATG8-containing autophagic membranes to study the function of each mATG8 protein in autophagy or selective autophagy, using each identified selective mATG8-binding LIR motif. To this end, we replaced the LIR1/2 and LIR3 motifs within RavZ(ΔCA)-GFP (termed gProbe, g: GFP) with selective mATG8-binding LIR motifs (gProbe-X, X: the protein name from which the LIR motif originated), as described previously [30]. Each probe, which contained two identical LIR motifs and an MT domain, was co-expressed with each of the mRFP-mATG8 proteins in HKO cells (Figure 4A). At 24 h after transfection, the normalized A:C ratio was quantified in the HKO cells treated with 100 nM BafA1 in EBSS for 2 h (Table S2 and Figures 4B–E).

Among the selective LIR motifs that we characterized, LIR(Sp) was selective for GABARAPL2 (Figures 2A,B). Therefore, we first tested whether gProbe-Sp selectively localized to GABARAPL2-containing autophagic membranes. Indeed, gProbe-Sp was mostly localized to GABARAPL2-containing autophagic membranes; however, it was also localized to other types of mATG8-containing autophagic membranes, indicating that gProbe-Sp can detect all types of mATG8-containing autophagic membranes at different levels (Figure 4B and Table S2). L397 in the LIR(Sp) hydrophobically interacted with W62 in GABARAPL2, which corresponds to F62 in GABARAP and GABARAPL1 (Figure 3B). Therefore, we considered L397 a good candidate mutation to generate a more selective GABARAPL2-binding LIR motif. We replaced L397 with I (LIR(Sp[I]) to generate gProbe-Sp(I), which selectively localized to GABARAPL2-containing autophagic membranes (Figures 4C and Table S2), suggesting that gProbe-Sp(I) is a highly selective probe for GABARAPL2-containing autophagic membranes.

Next, we used gProbe-TP(T), gProbe-4B(T), and gProbe-BNIP3L-pm in our cellular assay. gProbe-TP(T) only localized to LC3C-containing autophagic membranes, whereas gProbe-4B(T) selectively localized to GABARAP and GABARAPL1-containing, but not to, other LC3 subfamily- or GABARAPL2-containing autophagic membranes (Figures 4D,E). These results indicate that gProbe-TP(T) and gProbe-4B(T) are specific probes for LC3C- or GABARAP and GABARAPL1-containing autophagic membranes, respectively. In contrast, Probe-BNIP3L-pm was strongly associated with GABARAPL1-containing autophagic membranes and other types of mATG8-containing autophagic membranes (Table S2). Further mutational analyses are necessary to identify probes that specifically detect GABARAPL1-containing autophagic membranes.

Finally, we confirmed LC3 subfamily- or GABARAP subfamily containing autophagic membrane targeting using previously developed gProbe-Fy or gProbe-St, respectively [30], in HKO cells (Table S2). Taken together, we developed gProbe-Sp(I), gProbe-TP(T), and gProbe-4B(T) as selective probes that bind to membrane-anchored GABARAPL2-, LC3C-, and GABARAP and GABARAPL1 on autophagic membranes in cells, respectively.

To further examine whether our selective LIR-based sensors could indeed detect autophagosomes at the ultrastructural level, we performed correlative light and electron microscopy (CLEM) analysis to probe positive spots of interest in whole-cell images with fluorescence light microscopy and then zoomed in for a closer look using electron microscopy. This dual examination provided valuable, complementary, and unique information regarding the cellular and ultrastructural components of endogenous mATG8-containing autophagosomes. To this end, rProbe-Fy (GFP in gProbe-Fy replaced by mRFP, r: mRFP) and gProbe-Sp(I) were co-transfected into WT HeLa cells to determine whether membrane-anchored LC3 subfamily- or GABARAPL2-selective probes detected the same or distinct autophagosomes. HeLa cells were treated with rapamycin (100 nM) in the presence of BafA1 (100 nM) for 2 h to maximize the number of autophagosomes. Both rProbe-Fy and gProbe-Sp(I) detected autophagosomes throughout the cell (Figures 4F–J). Notably, some autophagosomes were detected only by red signal, indicating only rProbe-Fy-containing autophagosome (Figure 4J). Taken together, these data suggest that our new selective LIR-based probes are useful for identifying autophagosomes containing distinct mATG8 subfamilies at cellular and ultrastructural levels.

### Development of enzymes that selectively delipidate mATG8–PE in autophagic membranes

**Figure 5.**
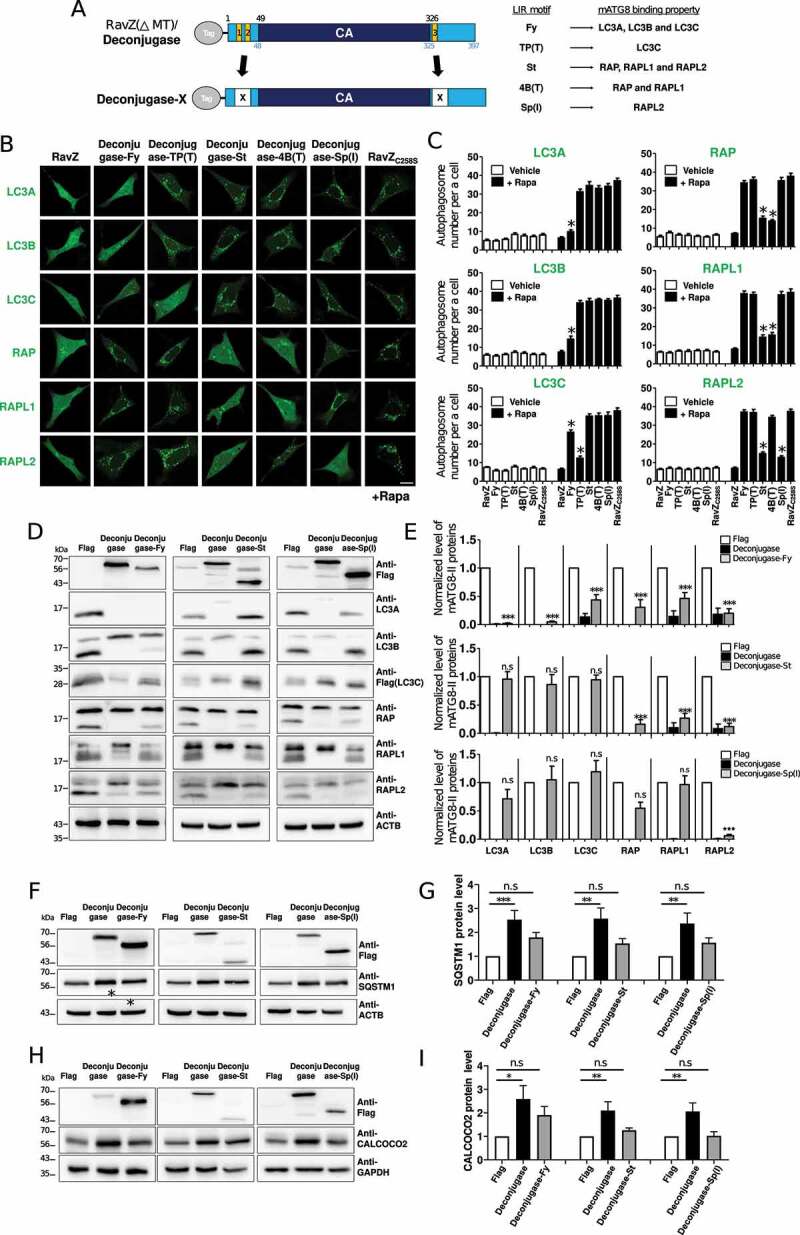
Generation of selective mATG8-delipidating deconjugase. (A) Schematic diagram of generation of deconjugase-X (×: Fy, TP[T], St, 4B[T], or Sp[I]) and its binding preferences. (B) Confocal images showing autophagosome formation of GFP-mATG8 protein co-expressed with RavZ proteins (RavZ, wild-type; RavZ_C258S_, catalytically inactive mutant) or deconjugase-X in mouse embryonic fibroblasts (MEFs) upon autophagy induction (100 nM rapamycin [Rapa], 4 h). Scale bar: 10 μm. (C) Bar graphs illustrate the autophagosome spot number for each cell. Values are presented as means + SEM (*n* ≥20 for each group). *P < 0.01 compared with RavZ_C258S_-expressing cells with one-way ANOVA in conjunction with the Newman – Keuls multiple comparison test. Rapa, rapamycin. (D – E) Representative western blots of four independent experiments of endogenous mATG8 proteins in HEK293T cells treated with 50 μM CQ for 4 h to accumulate autophagosome (D) and quantification data (E). As a control, Flag empty vector (Flag) was used. *** P < 0.001, n.S. not significant compared with each Flag-expressing cells, one-way ANOVA in conjunction with the Newman – Keuls multiple comparison test. Lysates of cells expressing 3xFlag-LC3C were used in the LC3C detection experiment. Data from representative experiments of three independent experiments are presented. (F-I) Deconjugases increased SQSTM1 and CALCOCO2 protein levels and SQSTM1 puncta size. Representative western blots of SQSTM1 (F) and CALCOCO2 (H) in deconjugase-X (X: Fy, St, Sp[I]) expressing HEK293T cells and their quantification data (G, I). As a control, Flag empty vector (Flag) was used. Values are presented as the mean + SEM (n ≥ 5). *P < 0.05, **P < 0.01 *** P < 0.001, n.s. not significant, one-way ANOVA in conjunction with the Newman – Keuls multiple comparison test.

To date, due to limitations in tools that can selectively inhibit or deplete each membrane-anchored mATG8 protein, information concerning the specific roles of the cytosolic and membrane-anchored forms of each mATG8 protein in autophagy and autophagy-independent pathways is scarce. Compared to mammalian ATG4B, which hydrolyzes the amide bond linking glycine and PE, RavZ hydrolyzes the amide bond between the C-terminal glycine residue and an adjacent aromatic residue, resulting in resistance to conjugation by the host machinery [31,32]. Therefore, to generate deconjugases that selectively remove PE from mATG8–PE in our study, we replaced LIR1/2 and LIR3 in 3xFlag-fused catalytically active RavZ protein (deconjugase) with two identical LIR(Fy), LIR(St), LIR(4B[T]), LIR(TP[T]), or LIR(Sp[I]), generating deconjugase-X: deconjugase-Fy targeted LC3A/B, deconjugase-St targeted the GABARAP subfamily, deconjugase-4B(T) targeted GABARAP and GABARAPL1, deconjugase-TP(T) targeted LC3C, and deconjugase-Sp(I) targeted GABARAPL2 (Figure 5A). We then monitored mATG8-containing autophagic membranes in rapamycin (100 nM)-treated mouse embryonic fibroblasts (MEFs) expressing deconjugase-X (X: Fy, St, TP[T], 4B[T], or Sp[I]) using GFP-mATG8 fluorescence. The expression of deconjugase-Fy selectively diminished LC3A/B-containing autophagic membranes (Figures 5B,C). Likewise, the expression of deconjugase-St, deconjugase-TP(T), deconjugase-4B(T), and deconjugase-Sp(I) selectively reduced GFP-GABARAP subfamily-containing, GFP-LC3C- containing, GFP-GABARAP- and GABARAPL1-containing, and GFP-GABARAPL2-containing autophagic membranes, respectively.

We detected endogenous levels of membrane-anchored mATG8 proteins in deconjugase-X (X: Fy, St, TP[T], 4B[T], or Sp[I])-expressing HEK293T cells treated with 50 μM chloroquine (CQ) impairing autophagosome-lysosome fusion for 4 h to increase mATG8-II level using western blot analysis to further determine enzyme specificity. Since the expression level of endogenous LC3C was too low to be detected by the anti-LC3C antibody, we expressed 3xFlag-LC3C and detected this using an anti-Flag antibody. Furthermore, we expressed deconjugases using a lentiviral vector to improve the expression levels. The expression of deconjugase-Fy strongly reduced the level of membrane-anchored LC3A and LC3B (LC3A-II and LC3B-II), but also significantly reduced that of membrane-anchored LC3C (LC3C-II) and membrane-anchored GABARAP subfamily (GABARAP-II, GABARAPL1-II and GABARAPL2-II) proteins, whereas deconjugase-St induced a selective reduction in the membrane-anchored form of GABARAP subfamily proteins, indicating selective delipidation (Figures 5D,E). Additionally, deconjugase-Sp(I) selectively downregulated membrane-anchored GABARAPL2 (GABARAPL2-II). In contrast, deconjugase-TP(T) and deconjugase-4B(T) non-selectively reduced membrane-anchored mATG8s (Figure S7). These results indicate that deconjugase-St and deconjugase-Sp(I) could be used to selectively delipidate the GABARAP subfamily and GABARAPL2, respectively. In the case of deconjugase-Fy, further improvement is required to selectively deplete the LC3 subfamily.

Finally, we examined the levels of SQSTM1 and CALCOCO2 as autophagic substrates in deconjugase-, deconjugase-Fy-, deconjugase-St-, or deconjugase-Sp(I)-expressing HEK293T cells by western blot analysis [18]. SQSTM1 and CALCOCO2 strongly accumulated in deconjugase-expressing HEK293T cells, indicating that deconjugase cleaved membrane-anchored mATG8 and inhibited autophagic degradation (Figures 5F,I). Conversely, the SQSTM1 and CALCOCO2 level were not significantly affected by deconjugase-Fy, deconjugase-St, or deconjugase-Sp(I) expression, suggesting that enzyme activity of deconjugase-Fy, deconjugase-St, or deconjugase-Sp(I) was not sufficient for inhibiting autophagic degradation of SQSTM1 and CALCOCO2.

### Membrane-anchored GABARAP subfamily proteins regulate TDP-25-mediated aggrephagy

We attempted to elucidate the specific roles of mATG8 proteins in aggrephagy, a type of selective autophagy [10], using our selective mATG8 LIR-based probes and deconjugases. Although several aggregate-prone proteins play roles in many human diseases, including neurodegenerative diseases, and may be cleared via aggrephagy, little is known about the differential roles of membrane-anchored LC3 or GABARAP subfamily proteins in aggrephagy.

**Figure 6.**
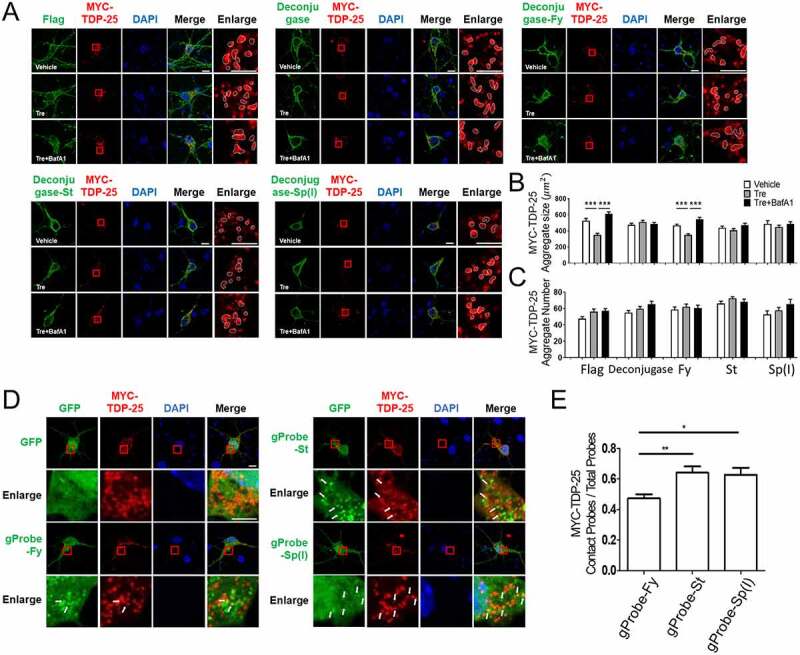
Regulation of TDP-25-mediated aggregates by selective mATG8 deconjugases. (A) Confocal images showing TDP-25-mediated aggregates in mouse cortical neurons transiently expressing deconjugase-X together with MYC-TDP-25. Scale bar: 10 μm. X: Fy, TP(T), St, 4B(T), or Sp(I). The neurons were incubated with trehalose (100 mM, 24 h) to activate autophagy. Mouse cortical neurons were treated with BafA1 (100 nM, 4 h) to block autophagosome and lysosome fusion. (B-C) Bar graphs show TDP-25 aggregate size (B) and number (C) per cell. Values are presented as means + SEM (*n* ≥17 for each group). ***P < 0.001, one-way ANOVA in conjunction with the Newman–Keuls multiple comparison test. (D) Confocal images showing cellular localization of TDP-25 aggregates with gProbe-X. GFP empty vector was used as the control. Scale bar: 10 μm. X : Fy, St, Sp(I). (E) The bar graph shows relative TDP-25 aggregates contacting the gProbes. Values are presented as means + SEM (*n* ≥11 for each group). *P < 0.05, ** P < 0.01, one-way ANOVA in conjunction with the Newman–Keuls multiple comparison test.

Thus, we investigated which mATG8 proteins were involved in the autophagic degradation of TDP-25 aggregates. TDP-25 is a pathogenic aggregate-prone 25-kDa C-terminal protein of TARDBP/TDP-43 that has been identified in protein inclusions in several neurodegenerative diseases, including frontotemporal dementia and ALS [33,34]. First, we transfected MYC-TDP-25 into cultured cortical neurons to verify whether MYC-TDP-25 aggregates were degraded by autophagy. TDP-25-positive aggregates were observed 24 h after transfection. When autophagy was activated with trehalose, an mTOR-independent autophagy inducer, the size of TDP-25-positive aggregates was significantly reduced, but their number was unaffected (Figures 6A–C). However, when autophagy was inhibited by BafA1 in neurons expressing MYC-TDP-25 upon autophagy induction, the size of TDP-25 aggregates increased, suggesting that the reduced size of TDP-25 aggregates might be due to the autophagic degradation of TDP-25.

Next, we investigated which mATG8 proteins were involved in aggrephagy by transfecting gProbe-Fy, gProbe-St, gProbe-4B(T), or gProbe-Sp(I) into cultured cortical neurons expressing MYC-TDP-25 to detect endogenous LC3 subfamily proteins, GABARAP subfamily proteins, GABARAP and GABARAPL1, and GABARAPL2. Neurons were treated with trehalose in the presence of BafA1 to maximize visual autophagosomes associated with or in contact with TDP-25 aggregates. gProbe-Fy, gProbe-St, gProbe-4B(T), and gProbe-Sp(I) were closely localized to the TDP-25 aggregates (Figures 6D,E). Remarkably, gProbe-St, gProbe-4B(T), and gProbe-Sp(I) were more closely localized to TDP-25 aggregates than gProbe-Fy, indicating that GABARAP subfamily proteins preferentially localized to TDP-25-positive aggregates (Figures 6D,E).

Moreover, we examined the size and number of TDP-25 aggregates in neurons expressing deconjugase, deconjugase-Fy, deconjugase-St, or deconjugase-Sp(I) after autophagy induction with trehalose in the presence or absence of BafA1 (100 nM, 2 h), to distinguish their differential roles in the regulation of TDP-25 aggregates by aggrephagy. We found that deconjugase expression failed to reduce the size of TDP-25 aggregates, indicating that deconjugase cleaved and inhibited all membrane-anchored mATG8 proteins, leading to cellular defects in aggrephagy (Figures 6A–C). Interestingly, deconjugase-St and deconjugase-Sp(I)-expressing neurons failed to reduce the size of TDP-25 aggregates compared to deconjugase-Fy-expressing neurons (Figure 6A–C). These data indicate that the membrane-anchored form of GABARAP subfamily proteins, but not LC3 subfamily proteins, regulates the degradation of TDP-25 aggregates during aggrephagy.

**Figure 7.**
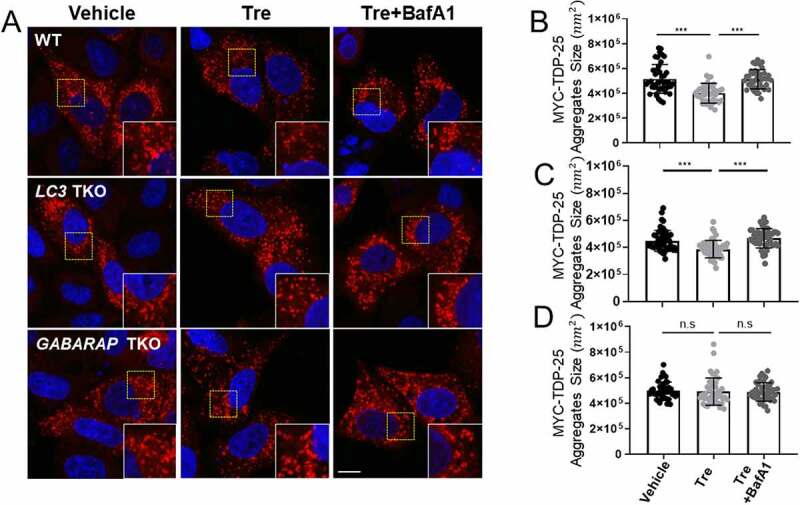
GABARAP subfamily proteins regulate TDP-25-positive aggregates. (A) Confocal images showing TDP-25-mediated aggregates in wild-type (WT), *LC3* triple knockout (TKO), or *GABARAP* TKO HeLa cells. Scale bar: 10 μm. The cells were incubated with trehalose (Tre; 100 mM, 24 h) to activate autophagy. Cells were treated with bafilomycin A_1_ (BafA1; 50 nM, 24 h) to block autophagosome and lysosome fusion. (B-D) Bar graphs show TDP-25 aggregates size per cell. Values are presented as means ± SEM (n ≥ 40 for each group). *** P<0.001, n.s. not significant, one-way ANOVA in conjunction with the Newman–Keuls multiple comparison test.

To further confirm whether the GABARAP subfamily regulates cellular degradation of TDP-25 aggregates during aggrephagy, we expressed MYC-TDP-25 in WT, *LC3* triple knockout (TKO), and *GABARAP* TKO HeLa cells, and examined the size of TDP-25 aggregates after autophagy induction with trehalose in the presence or absence of BafA1 (100 nM) for 2 h. As shown in Figures 7A–D, the size of TDP-25 aggregates was significantly reduced by autophagy induction in WT and *LC3* TKO cells, but not in *GABARAP* TKO cells. This reduction is mediated by autolysosomes, because the inhibition of autophagy and lysosome fusion completely blocked this reduction (Figure 7). Taken together, our data strongly suggest that membrane-anchored GABARAP subfamily proteins, but not LC3 subfamily proteins, are involved in the cellular degradation of TDP-25 aggregates during aggrephagy.

## Discussion

LIR-based sensors have been developed to detect endogenous LC3/GABARAP subfamily proteins [15,35,36]. Most binding experiments with ATG8 proteins have been performed with cytosolic unlipidated/free mATG8 proteins. However, the mATG8 proteins frequently perform their function on autophagic membranes in the membrane-anchored/lipidated form. There is limited knowledge regarding the binding of mATG8s to autophagic membranes. In this study, we used cellular assays to determine cytosolic and autophagic membrane-anchored mATG8 proteins binding in cells. We improved the previous version of LIR-based autophagosome sensors by introducing highly selective LIR motifs for LC3C, GABARAP and GABARAPL1, and GABARAPL2. These improved sensors can detect different sets of autophagic membrane-anchored mATG8 proteins of live cells. For example, gProbe-TP(T) detects LC3C-containing autophagic membranes in WT HeLa cells but not in *ATG5* or *ATG7* KO HeLa cells or MEF cells, in which LC3C is not expressed (Figure S6B), thereby monitoring LC3C-containing autophagic membranes in live cells. Therefore, our LIR-based LC3- or GABARAP subfamily selective sensors will likely provide novel insights into the differential functions of mATG8 proteins by monitoring their cellular localization on autophagic membranes at different stages of macroautophagy/selective autophagy or nonconventional autophagy associated with membrane-anchored LC3/GABARAP subfamily proteins.

Although the specific roles of each mATG8 protein in selective autophagy are largely unknown, ATG8 is recruited together with cargo into autophagosomal membranes via interactions with autophagy receptors to facilitate cargo degradation [37]. Most autophagy receptors possess a LIR motif that allows their direct binding to LC3, whereas most autophagy receptor proteins have GABARAP-specific LIRs [38]. Therefore, it has been proposed that LC3 subfamily proteins are essential for cargo recruitment during selective autophagy [7]. However, in these studies, the specific roles of the cytosolic and membrane-anchored forms of each mATG8 during macroautophagy/selective autophagy could not be distinguished. Many studies have used knockdown or knockout systems for ATG8 proteins or ATG8 conjugation-deficient cells to study the function of mATG8s that lead to the depletion of both soluble (cytosolic) and membrane-anchored mATG8 proteins or inhibit the lipidation of all mATG8 proteins. To our knowledge, this is the first report on the use of irreversible selective mATG8 deconjugases consisting of ATG8-selective LIR motifs and the catalytic domain of RavZ to investigate the functional significance of membrane-anchored mATG8 proteins in several types of autophagy. The use of selective deconjugases revealed that endogenous GABARAP subfamily protein-containing autophagosomes preferentially localize to and contact TDP-25 aggregates. More importantly, GABARAP proteins, including GABARAPL2 in autophagosomes (membrane-anchored GABARAP proteins), regulate the cellular degradation of TDP-25 aggregates in an autophagy-dependent manner (Figure 6). Membrane-anchored LC3A/B may not be involved in the cellular degradation of TDP-25 aggregates, although some LC3A- and LC3B-positive autophagosomes were associated with TDP-25 aggregates. The data we obtained using *LC3* TKO or *GABARAP* TKO cells and our novel deconjugases strongly support our finding that membrane-anchored GABARAP family proteins regulate aggrephagy associated with TDP-25 aggregates (Figures 6 and 7). A study using m*ATG8*-knockout HeLa cells showed that GABARAP subfamily proteins regulate parkin-mediated mitophagy, a form of selective autophagy, whereas LC3 subfamily proteins mediate basal autophagy [23]. Harper et al. investigated the crucial roles of GABARAP subfamily proteins in selective autophagy, reporting that LC3 subfamily members were not involved in all steps of selective autophagy [39]. These results are consistent with our data, suggesting that membrane-anchored GABARAP subfamily proteins contribute to selective autophagy. Additionally, our cellular assays revealed that many LIR motifs selectively bind to GABARAP subfamily proteins and not to LC3A/B (Table 1). This might be because many different LIR-containing autophagy receptors or other autophagy machinery proteins, including fusion components, selectively bind to GABARAP subfamily proteins to regulate their selective targeting or autophagic degradation. However, further detailed cellular and molecular approaches are necessary to elucidate the exact mechanism that regulates the selective interaction of each GABARAP subfamily protein and autophagy component.

In conclusion, our membrane-anchored LC3- and GABARAP-selective LIR-based sensors and irreversible selective deconjugases for each autophagosome-bound mATG8–PE help elucidate the cellular localization and selective functions of these membrane-anchored mATG8 proteins in conventional and nonconventional autophagy.

## Materials and methods

### DNA constructs

All primers used are listed in Table S3. The sequence encoding 3xNLS was generated by polymerase chain reaction (PCR) amplification of C1-pEGFP-NUC vectors (Addgene, 58468; deposited by Dyche Mullins Lab) and inserted into an N3-mRFP vector [36] to generate N3-mRFP-3xNLS using the restriction enzyme set *Xho*1-*Not*1. All LIRs, including the mutant LIRs used in these experiments, were amplified by extension PCR without a template using three long primers of 45 to 50 bp (containing complementary sequences overlapping each other by 18 bp) and then inserted into N3-EGFP and N3-mRFP-3xNLS vectors using the restriction enzyme set *Hind*III-*Kpn*1. We mostly used an extended LIR motif with 10 *N*-terminal amino acids and 11 C-terminal amino acids, in addition to the core LIR motif sequence (W/F/Y)-X-X-(L/I/V), because the *N*-terminal and C-terminal amino acids may also contribute to the binding with mATG8 [8,18]. Mutagenesis of the LC3 or GABARAP subfamily was amplified by PCR using mutant primers for each LC3/GABARAP subfamily member and inserted into pcDNA3.1-EGFP or mRFP vectors [36] using the restriction enzyme *BamH*I-*Apa*1. The region encoding RavZ or RavZ^C258S^ was generated by PCR amplification of pcDNA3.1(−)-Flag-RavZ or RavZ^C258S^ vectors and inserted into the C1-3xFlag vector to generate C1-3xFlag-RavZ or RavZ^C258S^ using the restriction enzyme *Bgl*II-*Apa*1 [40]. The C1-3xFlag-RavZ(ΔMT) (deconjugase) construct was created by replacing the membrane-targeting domain-containing catalytic domain with a catalytic domain in the C1-3xFlag-RavZ vector. Additionally, N3-EGFP-RavZ(ΔCA)_X_ (gProbe-X) or C1-3xFlag-RavZ(ΔMT)_X_ (deconjugase-X) chimeras were created by replacing the RavZ LIR motifs with the LIR motifs of another protein, amplified using primers and then inserted into each vector. In this study, we used GST-LC3A, GST-LC3B, GST-LC3C, GST-GABARAP, GST-GABARAPL1, GST-GABARAPL2, 3xFlag, and the previously described DNA constructs GFP-LC3A, GFP-LC3B, GFP-LC3C, GFP-GABARAP, GFP-GABARAPL1, and GFP-GABARAPL2 [36].

### Cell culture, transfection, confocal microscopy, and drug treatment

All cells used in the experiment were cultured in Dulbecco’s modiﬁed Eagle’s medium (Cytiva lifesciences, SH30262.01) supplemented with 10 or 15% (v:v) fetal bovine serum (Gibco, 26140079) and 1% (v:v) penicillin/streptomycin (Gibco, 15140122) in an incubator with 5% CO_2_ at 37°C. Generation of Hexa m*ATG8* KO (HKO) HeLa, *LC3* TKO and *GABARAP* TKO cells was performed as previously described [23]. All cells, including *ATG5*- or *ATG7*-knockout HeLa cells, were seeded in a sticky-slide eight-well system (Ibidi, 80826) to obtain 40–60% confluent growth on the day of imaging. Cells were transfected with plasmid DNA constructs using calcium phosphate (Takara Bio, 631312) or Lipofectamine 2000 (Thermo Fisher Scientific, 11668027) 24 h before imaging. The relative amount of each construct was empirically determined based on the relative expression of each combination of constructs. HKO cells were treated with 100 nM bafilomycin A_1_ (Sigma-Aldrich, B1793) in Earle’s balanced salts solution (EBSS; Sigma-Aldrich, E3024) for 2 h to accumulate autophagosomes in cells. MEF was treated with 200 nM rapamycin (Sigma-Aldrich, R0395) in Dulbecco’s modiﬁed Eagle’s medium (DMEM) for 3 h to induce autophagy. The cells were observed under an inverted Zeiss LSM-700 scanning laser confocal microscope operated with the ZEN software. The laser excitation and spectral detection windows for ﬂuorochromes were 488 nm (508–543 nm) for GFP and 561 nm (578–649 nm) for mRFP. Appropriate GFP (500–550 nm) and mRFP (575–625 nm) emission ﬁlters were used for the sequential imaging of each ﬂuorescent protein.

Primary cortical neurons were isolated from the Institute of Cancer Research E16 mice. Dissected brain cortex was incubated at 37°C with 0.25% trypsin (Thermo Fisher Scientific, 25200056) to dissociate the cells. The cells were cultured on poly L-lysine-coated plates (Sigma-Aldrich, P9155) in minimum essential medium (MEM; Gibco, 11095–080) supplemented with 10% (v:v) fetal bovine serum, 2 mM L-glutamine, 0.45% (v:v) D-(+) glucose, and 1% (v:v) penicillin-streptomycin. After cells were attached to the plate, the MEM was replaced with Neurobasal medium (Gibco, 21103–044) supplemented with B-27, 2 mM L-glutamine, and 1% (v:v) penicillin-streptomycin and incubated in 5% CO_2_ at 37°C. Cells were transfected with a plasmid DNA construct using Lipofectamine 2000. Neurons were incubated with trehalose (Sigma-Aldrich, T9449;100 mM, 24 h) to activate autophagy. HEK293T cells (ATCC, CRL-3216) and mouse cortical neurons were treated with BafA1 (100 nM, 4 h) or CQ (Sigma-Aldrich, C6628; 50 µM, 4 h) to block autophagy.

## Quantitative analysis of N:C or A:C ratios

To express the quantitative ratio of the nuclear:cytosol (N:C) fluorescence intensities, the average nuclear and cytosolic fluorescence intensities of at least five randomly selected points in the nucleus and cytosol in a single cell were measured using the ImageJ software. Similarly, the quantitative N:C ratio of at least 20 randomly selected cells was analyzed. The obtained values were normalized to the values calculated for mRFP-3xNLS-expressing cells (Figure 1). We also confirmed that the expression of mRFP-3xNLS lacking the LIR motif did not affect the N:C distribution of GFP-mATG8(GA). To calculate the ratio of autophagosome:cytosol (A:C) fluorescence intensities, autophagy flux was induced and blocked in HKO cells by treatment with 100 nM bafilomycin A_1_ in Earle’s balanced salt solution (EBSS) for 2 h. Cells were then fixed in 4% PFA (Cepham Life Sciences, 66311). The average value of autophagosome or cytosolic fluorescence intensity was obtained from at least five randomly selected points in autophagosomes or the cytosol of a single HKO cell using ImageJ. Similarly, the quantitative A:C ratio of at least 20 randomly selected cells per experiment was obtained from three independent experiments. All statistical data were calculated and plotted using GraphPad Prism version 6.

### Spot number analysis

To detect a reduction in mATG8-positive autophagosomes by enzyme activation of RavZ in autophagy-induced cells, we counted the number of spots over a certain field size in a single cell using ImageJ software. The cell image was changed to an 8-bit image and then inverted. The background was removed to ensure that only the spots remained visible. Finally, we counted the number of spots using the “Analyze the Particle” function of ImageJ. A minimum of 20 cells were quantified using this approach. All statistical data were calculated and plotted using GraphPad Prism version 6.

## GST affinity-isolation assay

For the GST affinity-isolation assay, HEK293T cells were transfected with plasmid DNA encoding LIR motif(x)-GFP constructs using calcium phosphate transfection. After transfection, the cells were washed with phosphate-buffered saline (PBS; Sigma-Aldrich, P3813), harvested, lysed in GST affinity-isolation buffer solution (50 mM Tris-HCl, pH 7.5 [Roche, 10812846001], 150 mM NaCl [Sigma-Aldrich, S9888], 2 mM ethylenediaminetetraacetic acid [EDTA; Sigma-Aldrich, E9884], 1% Triton X‐100 [Sigma-Aldrich, T8787], and a protease inhibitor cocktail [Roche, 11836170001]), and cleared by centrifugation at 13,000 x g for 20 min. The cell lysates were incubated overnight with purified GST-mATG8 proteins and glutathione-conjugated agarose beads (Sigma-Aldrich, G4510) at 4°C. The following day, the samples were washed three to five times with the same GST affinity-isolation buffer solution at 4°C and the remaining supernatant was removed. The samples were resuspended in sodium dodecyl sulfate-polyacrylamide gel electrophoresis (SDS-PAGE) sample buffer, immediately boiled, and analyzed by SDS-PAGE with Coomassie Brilliant Blue (Sigma-Aldrich, 1154440025) staining.

### Co-immunoprecipitation (co-IP)

For transient transfection, HEK293T cells were plated at a density of 5–7 × 10^5^ cells/well in six-well plates and cultured for 24 h. The cells were transfected with plasmid DNA sets each encoding LIR motif(x)-GFP and 3xFlag-mATG8s using calcium phosphate and incubated for 24 h. For Flag immunoprecipitation, the transfected HEK293T cells were washed with PBS, harvested, and lysed with EDTA lysis buffer solution (1% Triton X-100; 50 mM Tris-HCl, pH 7.5; 150 mM NaCl; 5 mM EDTA; protease inhibitor cocktail). The cell lysates were incubated overnight with 50 μl (bead volume) of mouse anti-Flag M2 antibody-conjugated beads (Sigma-Aldrich, A2220) at 4°C. The beads were subsequently washed three times with lysis buffer. The immunoprecipitates were eluted by adding 2 μg/ml of 3xFlag peptides (Sigma-Aldrich, F4799) and resuspended in SDS-PAGE sample buffer, boiled, and analyzed by SDS-PAGE.

### Western blot

Samples obtained from the common cell lysate and GST affinity-isolation assay or immunoprecipitation assay were separated via SDS-PAGE, transferred to polyvinylidene difluoride membranes, blocked using a blocking buffer (5% skimmed milk powder [Sigma-Aldrich, M7409] in TBST [150 mM NaCl; 50 mM Tris-HCl, pH7.5; 0.1% Tween 20 {Sigma-Aldrich, P1379}]) for 1 h at room temperature, and incubated overnight with primary antibodies at 4°C. After three washes, the membranes were incubated with secondary antibodies conjugated with horseradish peroxidase (HRP) for 1 h. The signals were visualized using the WesternBright ECL solution (GenDEPOT, W3680–010). Flag (Sigma-Aldrich, F1804; 1:10,000), GFP (Santa Cruz Biotechnology; sc-9996, 1:10,000), LC3A (Cell Signaling Technology, 12741; 1:1,000), LC3B (Cell Signaling Technology, 2775; 1:1,000), GABARAP (Cell Signaling Technology, 13733; 1:1,000), GABARAPL1 (Genetex, GTX132644; 1:500), GABARAPL2 (Genetex, GTX132666; 1:500), SQSTM1/p62 (Abnova, H00008878-M01; 1:100,000), CALCOCO2/NDP52 (Cell Signaling Technology, 60732; 1:1,000), GAPDH (NKMAX, ATGA0592; 1:10,000), and ACTB/β-actin (Cell Signaling Technology, 4967; 1:10,000) antibodies were used. The secondary antibodies were HRP-conjugated mouse anti-rabbit (Santa Cruz Biotechnology, sc-2357; 1:10,000) and HRP-conjugated mouse IgG kappa binding protein (Santa Cruz Biotechnology, sc -516,102; 1:10,000).

### Correlative light and electron microscopy

CLEM was performed as previously described [41]. Briefly, HeLa cells were cultured in culture dishes until 20–30% confluent and then transfected with mRFP-LC3A or -LC3B and EGFP-GABARAPL2 using Lipofectamine 2000. Next, the cells were treated with rapamycin (100 nM) in the presence of BafA1 (100 nM) for 2 h and imaged under a Ti-RCP confocal light microscope. The cells were fixed with 1% glutaraldehyde and 1% paraformaldehyde in 0.1 M cacodylate solution (pH 7.0) for 2 h at 4°*C*. Then, the fixed cells were washed with 0.1 M cacodylate solution, post-fixed with 2% osmium tetroxide for 1 h at 4°C and stained in 0.1 mg thiocarbohydrazide in 10 ml distilled water and en bloc in 1% uranyl acetate before dehydration through a graded ethanol series. Finally, the samples were embedded using the EMBed-812 embedding kit (Electron Microscopy Sciences, 14120). The embedded samples were sectioned at 60 nm using an ultramicrotome and viewed using a Tecnai G2 transmission electron microscope at 120 kV. Confocal micrographs were produced as high-quality images using the PhotoZoom Pro 8 software. The enlarged fluorescence images were fitted to electron micrographs using the ImageJ BigWarp program.

### Lentivirus production

To generate lentiviruses for infection, Lenti-X 293T (Takara Bio, 632180) cells were co-transfected with pLenti-EF1a-3xFlag-RavZ, wild type, FYCO1, STBD1, SpHfl1(I), psPAX2, and pMD2.G using Lipofectamine 2000. The culture supernatant was collected at 48 h and 72 h after transfection and passed through a 0.45-µm filter. The viral particles were concentrated by ultracentrifugation (22,600 x g for 3 h) and resuspended in Dulbecco’s phosphate-buffered saline (DPBS; Hyclone, SH30028.02).

### Immunocytochemistry

Transfected cells were washed with PBS, fixed with 4% PFA for 10 min, and permeabilized with 0.1% Triton X-100 for 10 min. Then, they were blocked with 3% bovine serum albumin (Sigma-Aldrich, A2153) for 1 h at room temperature before incubation overnight with anti-MYC or anti-Flag antibodies (Sigma-Aldrich, F1804) at 4°C. Then, they were incubated in fluorescent conjugated anti-mouse secondary antibodies (Jackson ImmunoResearch Laboratories, 715-165-150) for 2 h at room temperature. Finally, the cells on the glass slides were washed twice with 1× PBS and the slides were mounted with mounting medium (Vectashield, H-1200). The preparations were analyzed using an LSM 880 confocal laser scanning microscope.

### Plasmid constructions for crystallization and ITC

The human TP53INP2 LIR (residues 28–40) and fission yeast Hfl1 LIR (residues 386–409) were fused to the N terminus of human GABARAP and GABARAPL2 with F3S/V4T and W3S/M4T mutations, respectively, to promote crystallization. All genes were inserted downstream of the sequence encoding the human rhinovirus (HRV) 3C protease recognition site in the pGEX6P–1 vector (Cytiva, 28954648), except for the Hfl1 LIR-GABARAPL2 fusion protein, which was inserted upstream of the sequence encoding the HRV 3C protease site following the maltose-binding protein (*MBP*) gene of the pET15b vector (Merck, 69661-3CN). The plasmids were constructed using the NEBuilder HiFi DNA Assembly Master Mix (New England Biolabs, E2621) or In-Fusion HD Cloning Kit (Takara Bio, 639648). The gene for HRV 3C protease was inserted between the *GST* gene and BamHI site of the pGEX6P–1 vector, from which the HRV 3C recognition site was removed.

### Protein and peptide purification

All proteins were expressed in *E. coli* BL21 (DE3) cells. For protein purification, bacteria were cultured at 37°C until the OD_600_ of the culture reached 0.8–1.0. Then, the culture was supplemented with 100 µM IPTG (Nacalai Tesque, 19742–07), and further incubated overnight at 16°C. The bacterial pellets were resuspended in PBS and 5 mM ethylenediaminetetraacetic acid (EDTA) and sonicated for 10 min. After centrifugation, the supernatants were recovered and subjected to a GST-accepting resin (Nacalai Tesque, 09277–14). The resin was washed thrice with PBS and eluted with 10 mM glutathione (Nacalai Tesque, 17050–56) and 50 mM Tris at pH 8.0. The eluates were desalted with PBS using a Bio-Scale Mini Bio-Gel *P*-6 desalting column (Bio-Rad Laboratories, 7325312) and then digested overnight with HRV 3C protease, which was purified by ourselves as described below, at 4°C. Artificial glycine-proline sequences were retained at the N terminus of the gene product, except for the Hfl1 LIR-GABARAPL2 fusion protein that retained artificial L-E-V-L-F-Q sequences at the C terminus. All mutations were generated using PCR-based mutagenesis. The samples were subjected to GST-accept resin to remove the digested GST tags and the flow-through fractions were recovered. Peptides used for ITC experiments were synthesized by Cosmo Bio (LIR(TP[T]), the sequence is DGTLIIDLPDSY) or by BEX (LIR[Sp], the sequence is LQFEIDDEMEPLYNQAKQMRYGDY). Briefly, the peptides (10 mg) were dissolved in 300 µL of distilled water and 10 µL of ammonium hydrate. The proteins and peptides except for HRV 3C protease were purified by size-exclusion chromatography with 20 mM HEPES at a pH of 6.8 and 150 mM sodium chloride using a Superdex 200 prep grade column or Superdex 75 10/300 GL column. HRV 3C protease was purified by ion-exchange chromatography with buffer A (20 mM Tris, pH 8.0) and buffer B (20 mM Tris, pH 8.0; 1 M sodium chloride) using HiTrap DEAE FF column (Cytiva, 17505501). Purified HRV 3C protease was supplemented with 20% glycerol and 1 mM dithiothreitol and stored at −80°C until use.

### Crystallization and diffraction data collection

All crystallizations were performed at 20°C using the sitting-drop vapor-diffusion method by mixing protein and reservoir solutions in a 1:1 volume ratio. For crystallization of TP53INP2 LIR-GABARAP fusion protein, 11.79 mg/ml protein was mixed with 10% 2-propanol, 0.1 M sodium phosphate/citric acid at pH 4.2, and 0.2 M lithium sulfate. For crystallization of SpHfl1 LIR-GABARAPL2 fusion protein, 40.931 mg/ml protein was mixed with 8% polyethylene glycol 3000 (Hampton Research, HR2–604) and 0.1 M sodium citrate at pH 5.8. The crystals were soaked in cryoprotectant and frozen in liquid nitrogen. Cryoprotectants for SpHfl1 LIR-GABARAPL2 and TP53INP2 LIR-GABARAP were prepared by supplementing each reservoir solution with 25% 2-methyl-2,4-pentanediol and 33% glycerol, respectively. The flash-cooled crystals were maintained under nitrogen gas at −178°C during data collection. Diffraction data were collected using an EIGER X4 M detector attached to the beamline BL-1A at a wavelength of 1.1000 Å. Diffraction data were indexed, integrated, and scaled using energy-dispersive X-ray spectroscopy [42].

### Structure determination

The structures of the SpHfl1 LIR-GABARAPL2 and TP53INP2 LIR-GABARAP fusion proteins were determined by the molecular replacement method using the Phenix program [43]. GABARAP (PDBID:1GNU) and GABARAPL2 (PDBID:4CO7) structures were used as the search models. Crystallographic refinement was performed using Phenix and Coot programs [43,44]. All structural images were prepared using PyMOL Molecular Graphics System v2.0. Atomic coordinates and structure factors for LIR (Sp)-GABARALL2 fusion and LIR (TP)-GABARAP fusion were deposited in the Protein Data Bank (PDB, http://www.rcsb.org/) under PDB ID codes 7YO8 and 7YO9, respectively.

### Isothermal titration calorimetry

ITC experiments were performed using a MicroCal iTC200 calorimeter at 25°C with stirring at 1,000 rpm. SpHfl1 and TP53INP2 peptides (500 µM) were prepared as injection samples. The LC3 family and GABARAP subfamily proteins (50 µM) were prepared as cell samples. After a test injection of 0.4 µL, titration involved 18 injections of 2 µL of the injection samples at intervals of 120 s into the cell. The datasets obtained from titration of the peptides into cells filled with buffer were used as reference data to subtract the dilution heat. MicroCal Origin 7.0 software was used for data analysis. Thermal measurement data for the first test injection of syringe samples were removed from the analysis. Thermal titration data were fitted to a single-site binding model that was used to determine thermodynamic parameters, such as enthalpy, K_d_, and stoichiometry of binding (N). When the fit did not converge owing to weak interactions, N was fixed at 1.0. The error for each parameter represents the fitting error.

### Quantification and statistical analysis

All data are presented as mean ± SEM and were obtained from experiments performed in triplicate or higher. The Kolmogorov-Smirnov normality test was performed to check the Gaussian distribution of the group. For two-group comparisons, a two-tailed Student’s *t*-test was used. For multiple group comparisons, one-way ANOVA in conjunction with the Newman – Keuls multiple comparison test or the Kruskal – Wallis test followed by a Dunn multiple comparison test was performed as a parametric or non-parametric test, respectively. Statistical analysis was performed using GraphPad Prism 6 software. Statistical significance was set at P < 0.05.

## Supplementary Material

Supplemental Material
