# Supplementary material for: Development of new tools to study membrane-anchored mammalian Atg8 proteins
Source: Autophagy. 2022 Oct 17;19(5):1424–43. doi: 10.1080/15548627.2022.2132040 (PMC10240976; doi:10.1080/15548627.2022.2132040)
Supplement: Supplemental Material [file KAUP_A_2132040_SM5826.docx]

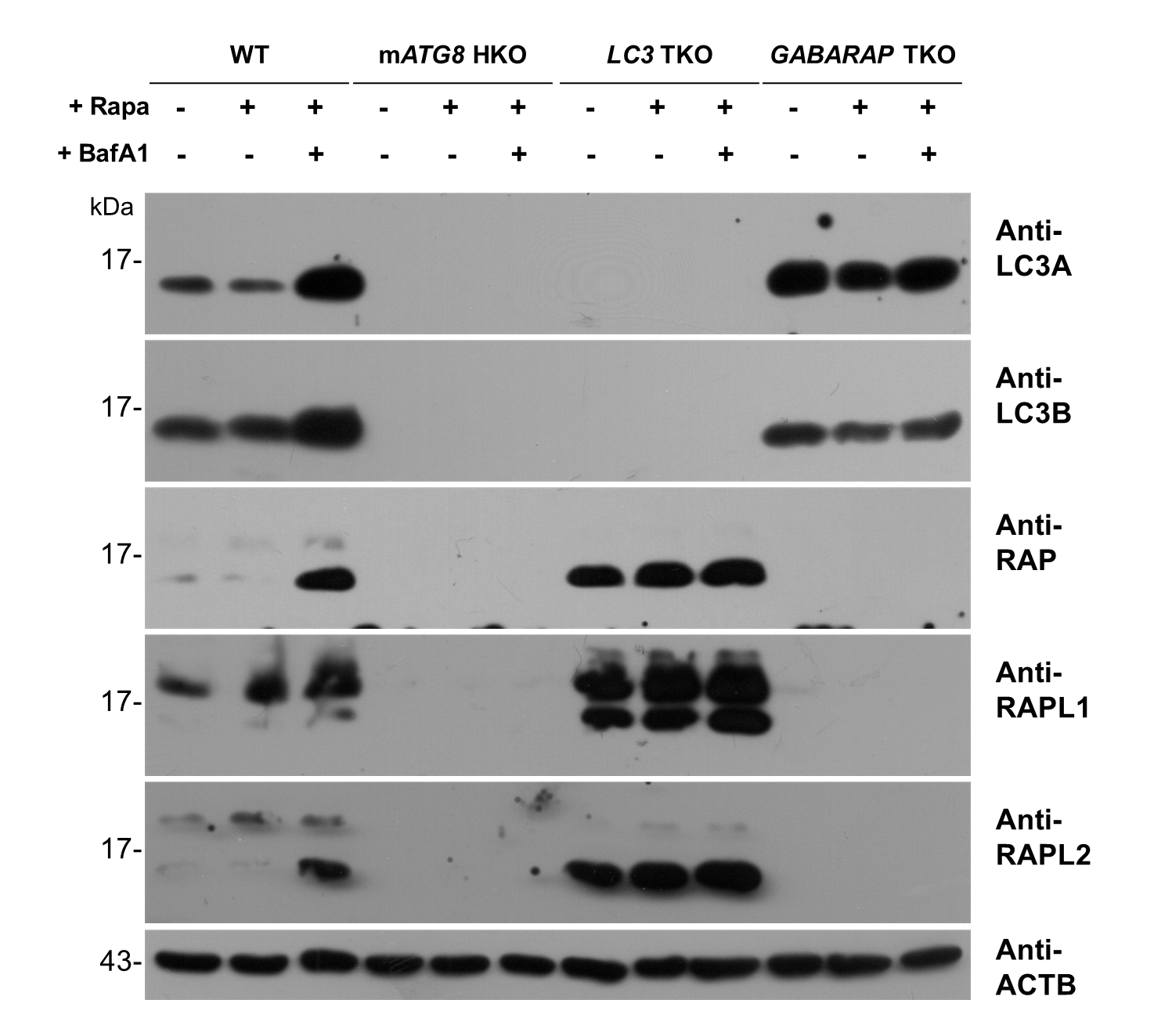


**Figure S1.** Immunoblotting of various HeLa cell lines including *LC3* TKO, *GABARAP* TKO, and hexa KO. Autophagic flux assay to verify the presence or absence of mATG8 in HeLa m*ATG8* hexa knockout (HKO) cells, *LC3* triple knockout (TKO), and *GABARAP* TKO. Wild-type HeLa (WT) cells and m*ATG8* HKO cells were prepared in an untreated group, + rapamycin (Rapa;100 nM, 2 h)-treated group, and +Rapa (100 nM, 2 h) and bafilomycin A_1_ (BafA1; 100 nM, 2 h)-treated group. Each mATG8 protein except LC3C was analyzed by western blot using the indicated anti-LC3A, -LC3B, -GABARAP, -GABARAPL1, and -GABARAPL2 and ACTB/β-actin antibodies.


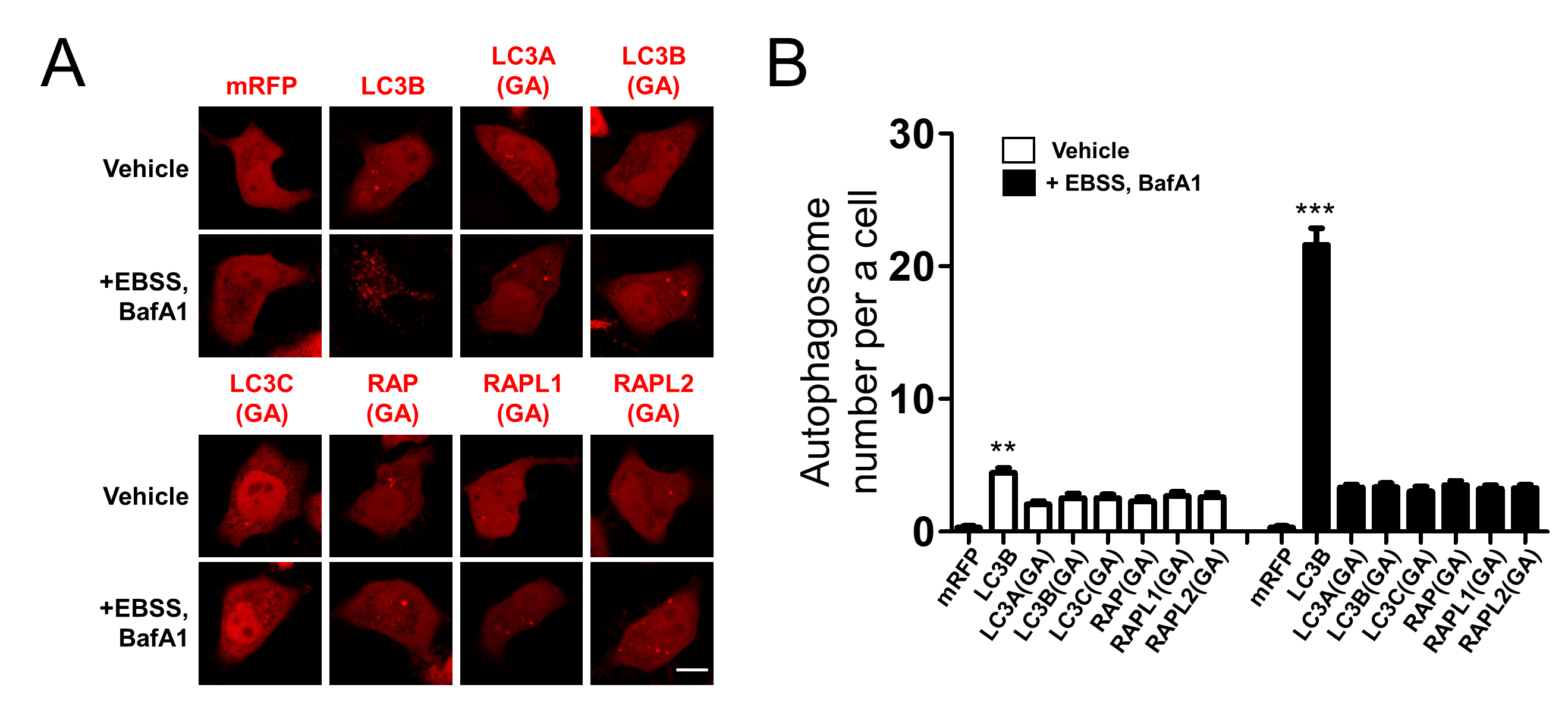


**Figure S2.** Diffusible distribution of unlipidated mATG8s in autophagy-induced HKO cells. (**A**) Cellular localization of mRFP, mRFP-LC3B, or mRFP-mATG8(GA) in HeLa m*ATG8* hexa knockout (HKO) cells upon vehicle treatment or autophagy induction (100 nM bafilomycin A_1_ [BafA1] in Earle’s Balanced Salts solution [EBSS] for 2 h, +EBSS, BafA1). Scale bar: 10 μm. (**B**) Quantification of the GFP-spot number per cell. Values are presented as means + SEM (*n* ≥20 for each group). ** P < 0.01, ***P < 0.001 compared with all other mRFP-mATG8(GA) expressing groups with one-way ANOVA in conjunction with the Newman–Keuls multiple comparison test.

**
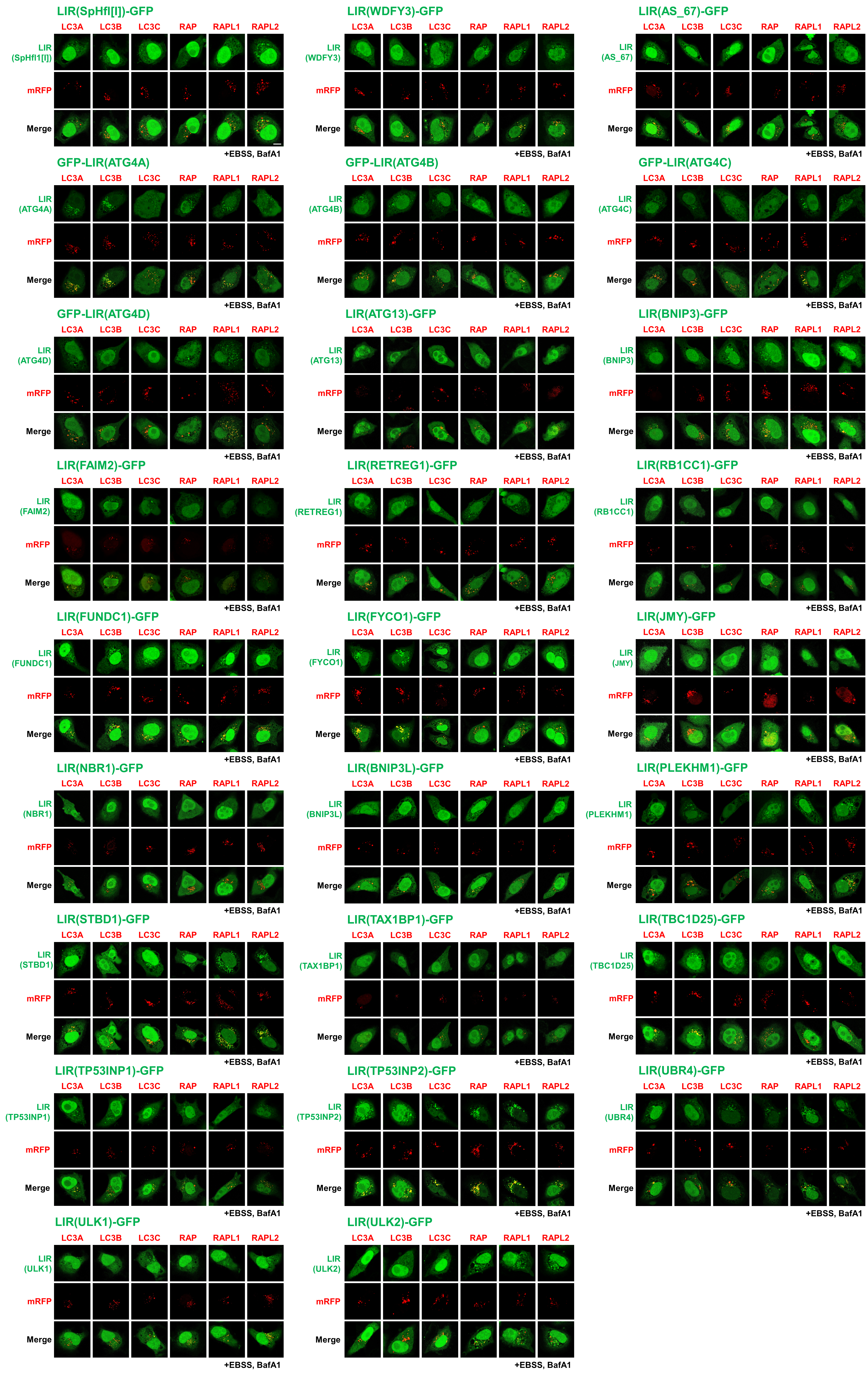
**

**Figure S3.** Image data showing cellular localization of LIR(X)-GFP as described in Table 1. Cellular localization of LIR(X)-GFP and each mRFP-mATG8 in HeLa m*ATG8* hexa knockout (HKO) cells upon autophagy induction (100 nM bafilomycin A_1_ [BafA1] in Earle's Balanced Salts solution [EBSS] for 2 h, +EBSS, BafA1) as shown in Table 1. Scale bar: 10 μm.


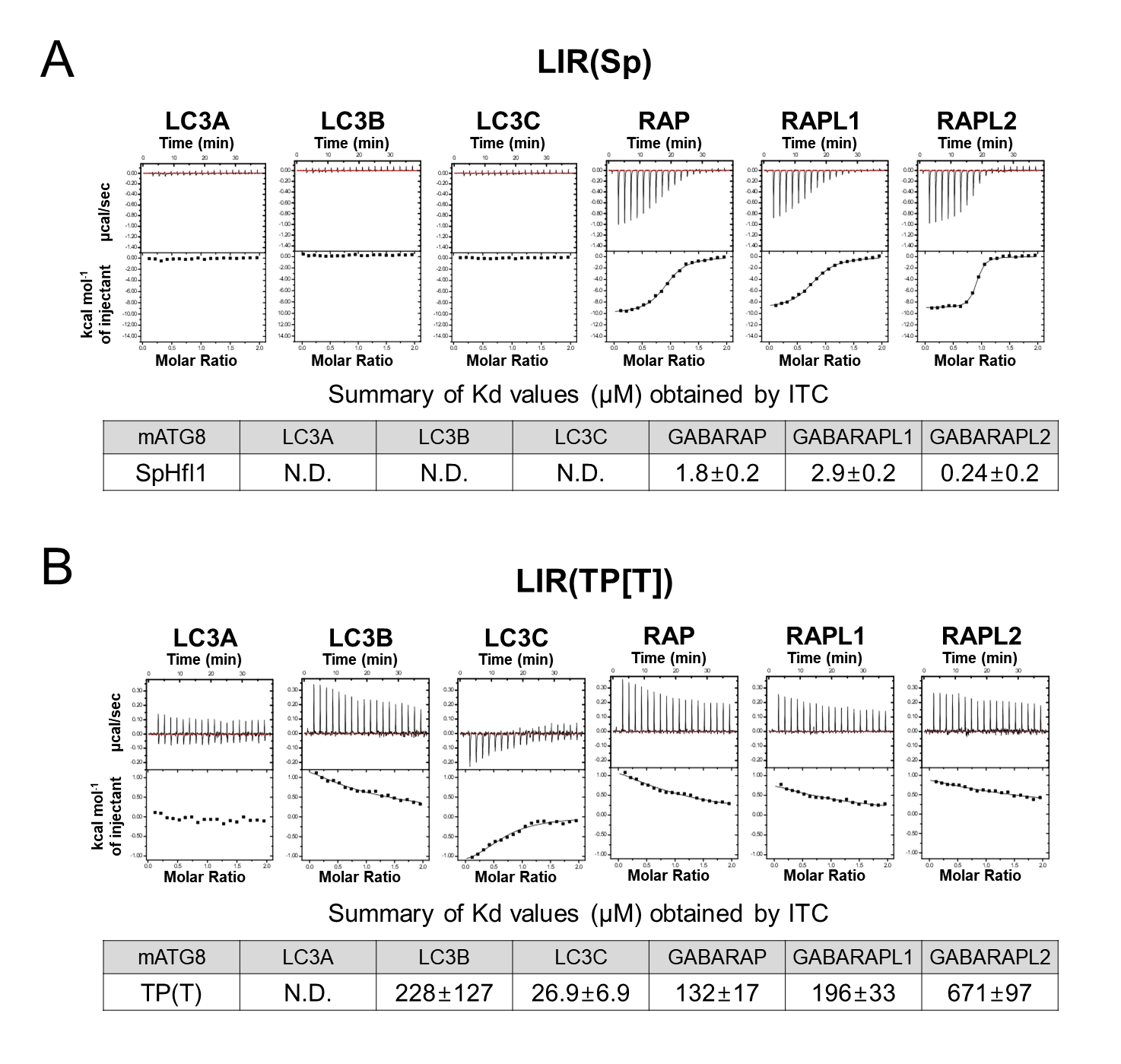


**Figure S4.** ITC analysis of LIR(Sp) selectively binding to GABARAPL2 and LIR(TP[T]) selectively binding to LC3C. (**A**) ITC data of mATG8 protein binding to with LIR(Sp). (**B**) ITC data of mATG8 protein binding to LIR(TP[T]).


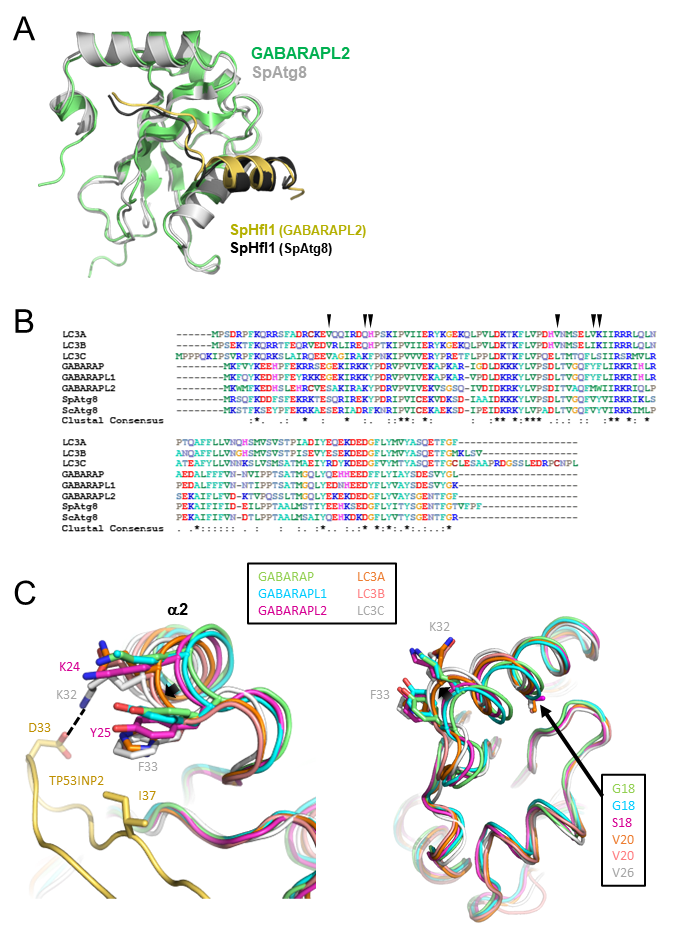


**Figure S5.** Residues responsible for specific interactions. (**A**) Structural comparison of SpHfl1 LIR bound to GABARAPL2 with that bound to SpAtg8 (PDBID 6AAF). The Cα atoms were used for superimposition. (**B**) Sequence alignment of mATG8 proteins and fungal Atg8 proteins. Arrowheads indicate the residues that were involved in LIR binding but not strictly conserved among mATG8s. (**C**) Structural comparison of the α2 helix of mATG8s. The Cα atoms were used for superimposition. The PDBIDs used were as follows: LC3A, 5CX3; LC3B, 5D94; LC3C, 3VVW; GABARAP, LIR(TP) complex (this study); GABARAPL1, 6HOI; and GABARAPL2, SpHfl1 LIR complex (this study).


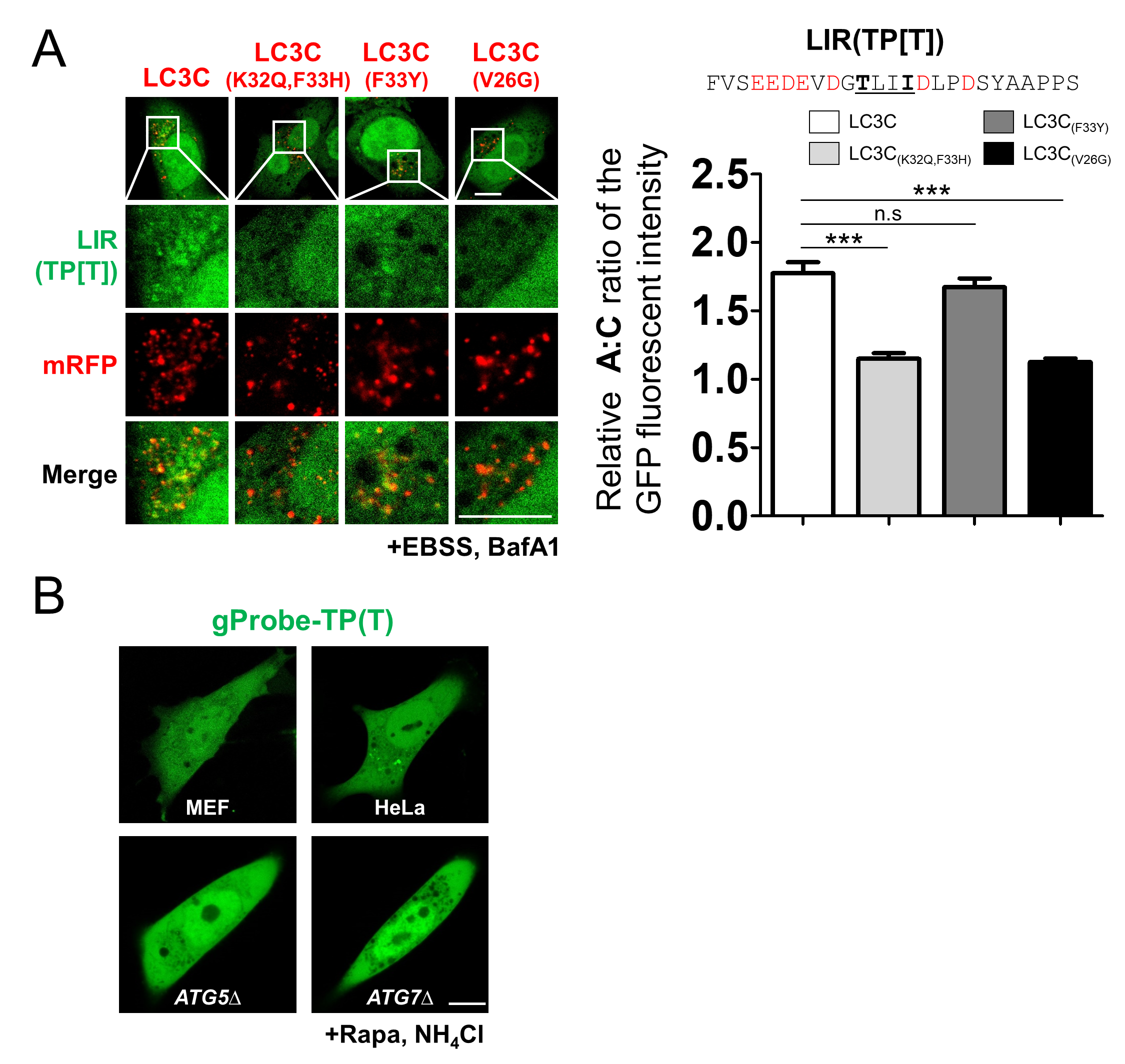


**Figure S6.** Characterization of LC3-selective LIR(TP[T]) motif. (**A**) Cellular localization of mRFP-LC3C, mRFP-LC3C(K32Q,F33H), mRFP-LC3C(F33Y), and mRFP-LC3C(V26G) co-expressed with LIR(TP[T])-GFP in HeLa m*ATG8* hexa knockout (HKO) cells (left) upon autophagy induction (100 nM bafilomycin A_1_ (BafA1) in Earle's Balanced Salts solution (EBSS) for 2 h, +EBSS, BafA1). Scale bar: 10 μm. Quantification of the relative autophagosome and cytosol (A:C) ratio of GFP fluorescence in cells (right). Values are presented as means + SEM (n ≥20 for each group). ***P < 0.001, n.s., not significant, one-way ANOVA in conjunction with the Newman–Keuls multiple comparison test. LIR(TP[T]), LIR(TP[T])-GFP. (**B**) Cellular localization of gProbe-TP(T) detecting LC3C-containing autophagic membranes in mouse embryonic fibroblast (MEF), HeLa, *ATG5*-knockour (KO) HeLa, and *ATG7*-KO HeLa cells upon autophagy induction (100 nM rapamycin [Rapa] + 10 mM NH4Cl for 2 h). Scale bar: 10 μm.


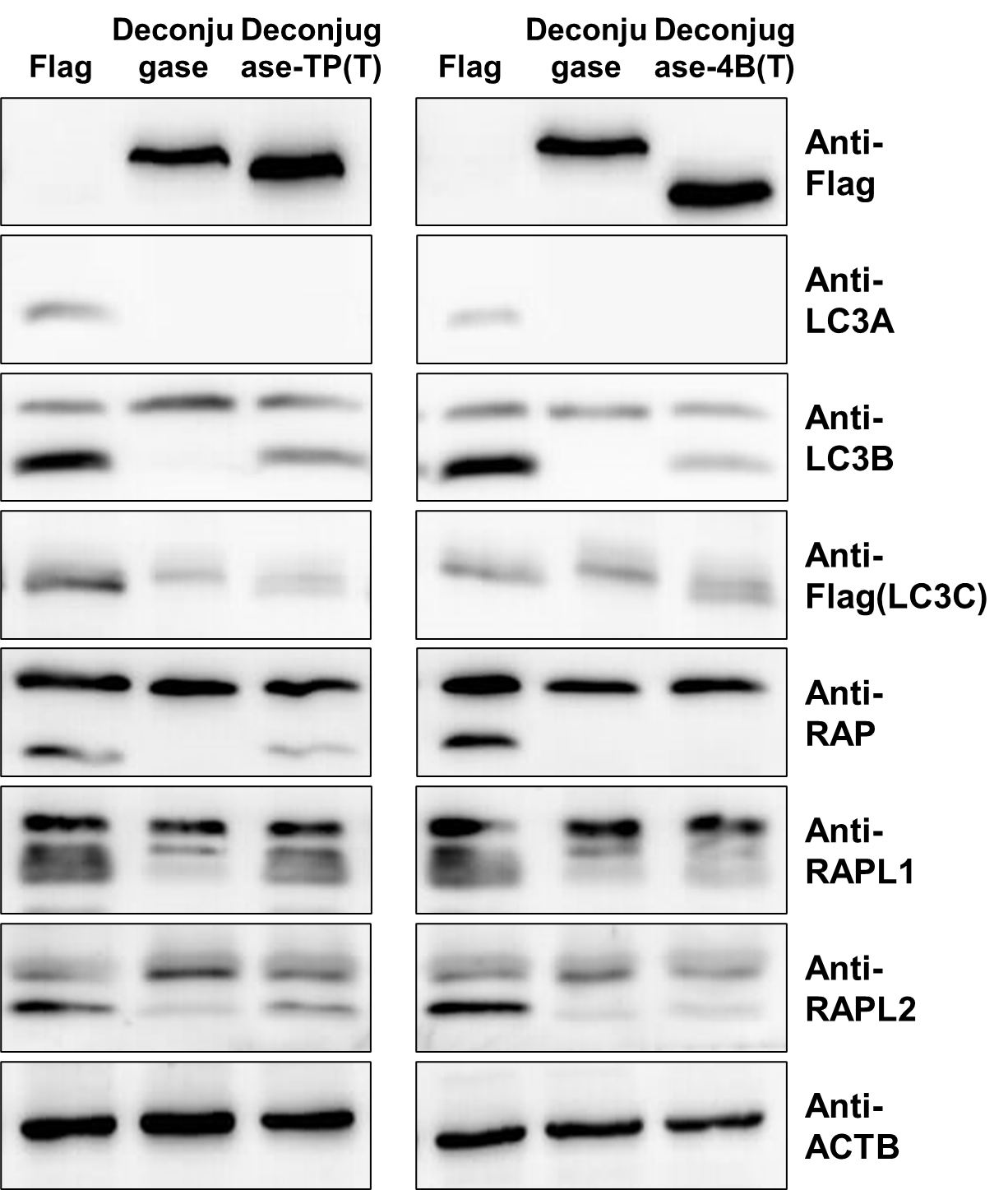


**Figure S7.** Non-selective delipidation of endogenous mATG8 by Deconjugase-TP(T) or Deconjugase-4B(T). Representative western blots of four independent experiments indicating expression of endogenous mATG8 proteins after expression of Deconjugase-TP(T) and Deconjugase-4B(T) in HEK293T cells to accumulate autophagosome (50 μM chloroquine for 4 h).

**Table S1.** Data collection and refinement statistics.

|  | LIR(Sp) - GABARAPL2 | LIR(TP) - GABARAP |
| --- | --- | --- |
| **Data collection** |  |  |
| Space group | *P*4_3_2_1_2 | *P*4_1_ |
| Cell dimensions |  |  |
| *a*, *b*, *c* (Å) | 45.85 45.85 152.58 | 46.39 46.39 75.40 |
| α, β, γ (°) | 90.0, 90.0, 90.0 | 90.0, 90.0, 90.0 |
| Resolution (Å) | 43.91-1.81 (1.87-1.81) | 46.39-1.75 (1.81-1.75) |
| *R*_sym_ or *R*_merge_ | 0.154 (1.469) | 0.099 (1.065) |
| *I* / σ*I* | 11.0 (1.79) | 10.8 (1.3) |
| Completeness (%) | 99.9 (99.2) | 99.7 (97.2) |
| Redundancy | 12.7 (12.6) | 7.0 (6.9) |
| CC1/2 | 0.997 (0.673) | 0.994 (0.764) |
|  |  |  |
| **Refinement** |  |  |
| Resolution (Å) | 43.91-1.81 | 46.39-1.75 |
| No. reflections | 15807 | 16075 |
| *R*_work_ / *R*_free_ | 0.178/0.206 | 0.194/0.216 |
| No. atoms |  |  |
| Protein | 1138 | 1083 |
| Ligand | 0 | 17 |
| Water | 149 | 77 |
| *B*-factors |  |  |
| Protein | 33.0 | 45.9 |
| Ligand |  | 77.9 |
| Water | 38.5 | 50.2 |
| R.m.s. deviations |  |  |
| Bond lengths (Å) | 0.006 | 0.007 |
| Bond angles (°) | 0.77 | 0.80 |

Values in parentheses are for highest-resolution shell.

**Table S2.** Summary of the normalized binding property of gProbe-LIR(X)-GFP probes to each membrane-anchored mATG8 in HKO cells treated with bafilomycin A_1_ (BafA1) in Earle’s Balanced Salts solution (EBSS) for 2 h.

| **gProbe** | **LC3/GABARAP family interaction** | | | | | |
| --- | --- | --- | --- | --- | --- | --- |
|  | **LC3A** | **LC3B** | **LC3C** | **GABARAP** | **GABARAPL1** | **GABARAPL2** |
| gProbe-Sp | **+** | **+** | **+** | **+** | **+** | **+++** |
|  | (1.479±0.043, N=31) | (1.537±0.036, N=30) | (1.682±0.069, N=30) | (1.897±0.046, N=30) | (1.87±0.087, N=31) | (5.126±0.2, N=22) |
| gProbe-Sp(I) | **-** | **-** | **-** | **-** | **-** | **+++** |
|  | (1.239±0.051, N=21) | (1.189±0.046, N=20) | (1.323±0.051, N=21) | (1.339±0.082, N=20) | (1.231±0.059, N=20) | (4.817±0.383, N=20) |
| gProbe-TP(T) | **-** | **-** | **+++** | **-** | **-** | **-** |
|  | (1.211±0.057, N=20) | (1.164±0.07, N=22) | (4.193±0.33, N=23) | (1.198±0.066, N=20) | (1.232±0.055, N=20) | (1.089±0.04, N=21) |
| gProbe-4B(T) | **-** | **-** | **-** | **+++** | **+++** | **-** |
|  | (1.385±0.059, N=20) | (1.382±0.056, N=20) | (1.337±0.074, N=20) | (4.783±0.187, N=20) | (4.873±0.131, N=20) | (1.329±0.049, N=20) |
| gProbe-Fy | **+++** | **+++** | **+** | **-** | **-** | **-** |
|  | (4.936±0.133, N=29) | (4.68±0.184, N=24) | (1.702±0.068, N=23) | (1.093±0.034, N=20) | (0.9741±0.034, N=20) | (1.042±0.041, N=20) |
| gProbe-St | **-** | **-** | **-** | **+++** | **+++** | **+++** |
|  | (1.259±0.045, N=22) | (1.255±0.051, N=20) | (1.312±0.06, N=20) | (6.25±0.405, N=22) | (5.956±0.463, N=22) | (5.527±0.53, N=20) |
| gProbe-ULK2 | **-** | **-** | **+** | **+++** | **+++** | **+++** |
|  | (1.134±0.039, N=21) | (1.317±0.057, N=21) | (1.72±0.109, N=22) | (5.393±0.268, N=22) | (5.123±0.16, N=22) | (4.111±0.255, N=22) |
| gProbe-BNIP3L-pm | **++** | **++** | **+** | **++** | **+++** | **++** |
|  | (2.415±0.127, N=15) | (2.241±0.127, N=15) | (1.948±0.131, N=18) | (2.585±0.165, N=15) | (4.527±0.175, N=15) | (2.31±0.126, N=15) |

The normalized A:C ratio: -1.4: - ; 1.4-2.0: +; 2.0-4.0: ++; 4.0: +++

**Table S3.** Primer sequences used for the experiments.

| Construct | Primer sequences (5’-3’) |
| --- | --- |
| LIR motif  (SQSTM1) | Forward: CCCAAGCTTGCCACCATGTCGGATAACTGTTCAGGAGGAGATGATGAC  Forward2: TCAGGAGGAGATGATGACTGGACCCATCTGTCTTCAAAAGAAGTGG  Reverse: GACGGTACCTTCACCTGTAGACGGGTCCACTTCTTTTGAAGACA |
| LIR motif  (SQSTM1m) | Forward: CCCAAGCTTGCCACCATGTCGGATAACTGTTCAGGAGGAGATGATGAC  Forward2: TCAGGAGGAGATGATGACGCCACCCATGCCTCTTCAAAAGAAGTGG  Reverse: GACGGTACCTTCACCTGTAGACGGGTCCACTTCTTTTGAAGAGG |
| LIR motif  (SpHfl1) | Forward: CGCCCAAGCTTCTCCAATTTGAAATAGACGATGAAATGGAACC  Forward2: AGACGATGAAATGGAACCATTATACAATCAAGCGAAACAGAT  Reverse: GACGGTACCATAATCACCATAACGCATCTGTTTCGCTTGATTG |
| LIR motif  (SpHfl1[I]) | Forward: CGCCCAAGCTTCTCCAATTTGAAATAGACGATGAAATGGAACC  Forward2: ATAGACGATGAAATGGAACCAATATACAATCAAGCGAAACAGATG  Reverse: GACGGTACCATAATCACCATAACGCATCTGTTTCGCTTGATTG |
| LIR motif  (TP53INP2) | Forward: CGCCCAAGCTTTTCGTGTCGGAGGAGGATGAAGTGGACGGCTGG  Forward2: GATGAAGTGGACGGCTGGCTCATCATTGACCTGCCGGACAGCT  Reverse: GACGGTACCGCTGGGTGGAGCCGCGTAGCTGTCCGGCAGGTCAA |
| LIR motif  (TP53INP2[T]) | Forward: CCCAAGCTTGCCACCATGTTCGTGTCGGAGGAGGATGAAGTGGACGGCA  Forward2: GATGAAGTGGACGGCACCCTCATCATTGACCTGCCGGACAGCT  Reverse: GACGGTACCGCTGGGTGGAGCCGCGTAGCTGTCCGGCAGGTCAA |
| LIR motif  (BNIP3L) | Forward: CGCCCAAGCTTCTGCCCCCGCCGGCCGGCCTCAACAGTTCCTGG  Forward2: GGCCTCAACAGTTCCTGGGTGGAGCTACCCATGAACAGCAGCA  Reverse: GACGGTACCGCCATTATCATTGCCATTGCTGCTGTTCATGGGTA |
| LIR motif  (BNIP3L-pm) | Forward: CCCAAGCTTGCCACCATGCTGCCCCCGCCGGCCGGCCTCAACGAAGAATGG  Forward2: GGCCTCAACGAAGAATGGGTGGAGCTACCCATGAACAGCAGC  Reverse: GACGGTACCGCCATTATCATTGCCATTGCTGCTGTTCATGGGTA |
| LIR motif  (ATG4A) | Forward: CCCAAGCTTGCCACCATGCAACTGGAGGAGTTTGATCTGGAGGAAG  Reverse: GACGGTACCCACACTCAGAATCTCAAAATCTTCCTCCAGATCAAACT |
| LIR motif  (ATG4B) | Forward: CCCAAGCTTGCCACCATGGAAAGATTCTTCGACTCAGAAGATGAAG  Reverse: GACGGTACCAAGGGACAGGATTTCAAAGTCTTCATCTTCTGAGTCGA |
| LIR motif  (ATG4B[T]) | Forward: CCCAAGCTTGCCACCATGGAAAGATTCTTCGACTCAGAAGATGAAG  Reverse: GACGGTACCAAGGGAAGTGATTTCAAAGTCTTCATCTTCTGAGTCGA |
| LIR motif  (ATG4C) | Forward: CCCAAGCTTGCCACCATGAAACAATTAAAAAGATTTAGCACGGA  Reverse: GACGGTACCAAGCAAGACAAACTCTTCCGTGCTAAATCTTTTT |
| LIR motif  (ATG4D) | Forward: CCCAAGCTTCTCAGGGCCAAACGCCCCAGCTCTGAGGACTTT  Reverse: GTAGGGCCCTTATAAAAACACAAAGTCCTCAGAGCTGGG |
| LIR motif  (ALFY) | Forward: CCCAAGCTTGCCACCATGGACCAGCTCAGTCTAGATGAGAAAGACG  Forward2: GTCTAGATGAGAAAGACGGCTTCATATTTGTGAACTATTCAGAGGG  Reverse: GACGGTACCCAGATGGGCTCTGGTCTGGCCCTCTGAATAGTTCACA |
| LIR motif  (AS_67) | Forward: CGCCCAAGCTTGCCACCATGAGCTTTACCATGTACGAA  Forward2: AGCTTTACCATGTACGAACCAGACCAACAGACCATAGTTATAG  Reverse: CGCGGTACCGCTTTCTATAACTATGGTCTGT |
| LIR motif  (ATG13) | Forward: CGCCCAAGCTTGGGGGCAGCAGTGGCAATACCCATGATGACTTT  Forward2: AATACCCATGATGACTTTGTTATGATAGACTTTAAACCAGCTT  Reverse: GACGGTACCAATGTCATCTTTAGAAAAAGCTGGTTTAAAGTCTA |
| LIR motif  (BNIP3) | Forward: CGCCCAAGCTTGGGATGCAGGAGGAGAGCCTGCAGGGCTCCTGG  Forward2: AGCCTGCAGGGCTCCTGGGTAGAACTGCACTTCAGCAATAATG  Reverse: GACGGTACCAACGCTGCCCCCGTTCCCATTATTGCTGAAGTGCA |
| LIR motif  (FAIM2) | Forward: CCCAAGCTTGCCACCATGGCCCCCACAGCGGTGCCTCTCCACCCTAGC  Forward2: GTGCCTCTCCACCCTAGCTGGGCCTATGTGGACCCCAGCAGCAGCTCC  Reverse: GACGGTACCACCGTTGTCATAGCTGGAGCTGCTGCTGGGGTC |
| LIR motif  (RETREG1) | Forward: CGCCCAAGCTTGAGGACACAGACACTGAAGAAGGTGATGACTTT  Forward2: GAAGAAGGTGATGACTTTGAACTACTTGACCAGTCAGAGCTGG  Reverse: GACGGTACCTTCACTCTCAATTTGATCCAGCTCTGACTGGTCAA |
| LIR motif  (RB1CC1) | Forward: CGCCCAAGCTTCCAGATAGTATTGATGCACATACGTTTGATTTT  Forward2: GCACATACGTTTGATTTTGAAACTATTCCCCATCCAAACATAG  Reverse: GACGGTACCTTGGTGAATAGTCTGTTCTATGTTTGGATGGGGAA |
| LIR motif  (FUNDC1) | Forward: CGCCCAAGCTTCCCCAAGACTATGAAAGTGATGACGACTCTTAT  Forward2: AGTGATGACGACTCTTATGAAGTGTTGGATTTAACTGAGTATG  Reverse: GACGGTACCCCACTGGTGTCTTCTTGCATACTCAGTTAAATCCA |
| LIR motif  (FYCO1) | Forward: CGCCCAAGCTTAGGCCACCGGACGACGCTGTGTTTGATATCAT  Forward2: CGCTGTGTTTGATATCATCACAGATGAGGAATTGTGCCAGATAC  Reverse: GACGGTACCTCAGGAGCCGGACTCCTGTATCTGGCACAATTCCT |
| LIR motif  (JMY) | Forward: CCCAAGCTTGCCACCATGTTCGCGCTGGAGGAGACGCTCGAGTCGG  Forward2: AGGAGACGCTCGAGTCGGACTGGGTGGCTGTGCGGCCCCATGTGTT  Reverse: GACGGTACCGTGTTTCTCGCGCTCGTCGAACACATGGGGCCGCACA |
| LIR motif  (NBR1) | Forward: CGCCCAAGCTTCAAAGTCAGTCCTCTGCTTCCTCAGAGGATTAC  Forward2: GCTTCCTCAGAGGATTACATCATCATCCTGCCTGAGTGCTTTG  Reverse: GACGGTACCCAGGGGGCGGCTGGTATCAAAGCACTCAGGCAGGA |
| LIR motif  (PLEKHM1) | Forward: CCCAAGCTTGCCACCATGCAGAAGGTCCGGCCTCAGCAGGAGGATG  Forward2: GGCCTCAGCAGGAGGATGAGTGGGTGAACGTGCAGTACCCAGACCA  Reverse: GACGGTACCCTCGGGGGGTTCCTCAGGCTGGTCTGGGTACTGCACG |
| LIR motif  (STBD1) | Forward: CGCCCAAGCTTAACAGTCAGGACCGGGTTGACCACGAGGAGTGG  Forward2: GTTGACCACGAGGAGTGGGAAATGGTGCCTAGGCACTCATCTT  Reverse: GACGGTACCCACACCAACATCCCCCCAAGATGAGTGCCTAGGCA |
| LIR motif  (TAX1BP1) | Forward: CGCCCAAGCTTACTATGGAAGATGAAGGAAATTCTGACATGTTA  Forward2: GGAAATTCTGACATGTTAGTGGTGACCACAAAAGCAGGCCTTC  Reverse: GACGGTACCCTCAATTTTCAACTCAAGAAGGCCTGCTTTTGTGG |
| LIR motif  (TBC1D25) | Forward: CGCCCAAGCTTCCCTCGGAGGACAGCCCATTGCTAGAAGACTGG  Forward2: CCATTGCTAGAAGACTGGGACATAATCAGCCCCAAAGATGTCA  Reverse: GACGGTACCCAACACGTCGGAGCCAATGACATCTTTGGGGCTGA |
| LIR motif  (TP53INP1) | Forward: CGCCCAAGCTTCCAGAATTCAATGAGAAAGAAGATGATGAATGG  Forward2: AAAGAAGATGATGAATGGATTCTTGTTGACTTCATAGATACTT  Reverse: GACGGTACCTGCTGAGAAACCAGTGCAAGTATCTATGAAGTCAA |
| LIR motif  (UBR4) | Forward: CCCAAGCTTGCCACCATGCAGGAACAGAGTGAGGTGGACCATGGAG  Forward2: GTGAGGTGGACCATGGAGATTTTGAGATGGTGTCTGAGTCGATGGT  Reverse: GACGGTACCATTTTCAGCTGTCTCCAGGACCATCGACTCAGACACC |
| LIR motif  (ULK1) | Forward: CGCCCAAGCTTAGCAAGGACTCTTCCTGTGACACAGACGACTTC  Forward2: TGTGACACAGACGACTTCGTCATGGTCCCCGCGCAGTTTCCAG  Reverse: GACGGTACCCTCAGCCACCAGGTCACCTGGAAACTGCGCGGGGA |
| LIR motif  (ULK2) | Forward: CGCCCAAGCTTAGCAAGAACTCTTCTTGTGACACGGATGACTTT  Forward2: TGTGACACGGATGACTTTGTTTTGGTGCCACACAACATCTCGT  Reverse: GACGGTACCATCACATGAGTGGTCTGACGAGATGTTGTGTGGCA |
| 3xNLS | Forward: AGATCTCGAGCTGATCCAAAAAAGAAGAGAAAG  Reverse: ATAAGAATGCGGCCGCTTATCCTACCTTTCTCTTCT |
| LC3A  (G120A) | Forward: CGCGGATCCATGCCCTCAGACCGGCCT  Reverse: CGTAGGGCCCTCAGAAGGCGAAGGTTTCCTGGG |
| LC3B  (G120A) | Forward: CGCGGATCCATGCCGTCGGAGAAGACC  Reverse: CGTAGGGCCCTTACACTGACAATTTCATGGCGAA |
| LC3C  (G126A) | Forward: CGCGGATCCATGCCGCCTCCACAGAAA  Reverse: TCCTCAAGGCTGCTCCCATCCCTGGGGGCTGCTGACTCCAGGCAGGCAAA  Reverse2: CGTAGGGCCCCTAGAGAGGATTGCAGGGTCTGTCCTCAAGGCTGCTCCCA |
| GABARAP  (G116A) | Forward: CGCCCAAGCTTATGAAGTTCGTGTACAAA  Reverse: CGTAGGGCCCTCACAGGGCGTAGACACTTTCGTCAC |
| GABARAPL1  (G116A) | Forward: CGCGGATCCATGAAGTTCCAGTACAAG  Reverse: CGTAGGGCCCTCATTTGGCATAGACACTCTCAT |
| GABARAPL2  (G116A) | Forward: CGCCCAAGCTTATGAAGTGGATGTTCAAG  Reverse: CGTAGGGCCCTCAGAAGGCAAAAGTGTTCTCTC |
| GABARAPL2  (W62A) | Forward: CGCCCAAGCTTATGAAGTGGATGTTCAAG  Reverse: GATCCTTTTCCTGATGATCGCCATGAACTGAGCCACAGT  Forward2: ACTGTGGCTCAGTTCATGGCGATCATCAGGAAAAGGATC  Reverse3: CACGCGTCGACTCAGAAGCCAAAAGTGTT |
| GABARAP  (F62W) | Forward: CGCCCAAGCTTATGAAGTTCGTGTACAAA  Reverse: AATTCGCTTCCGGATCAACCAGTAGAACTGACCAACTGT  Forward2: ACAGTTGGTCAGTTCTACTGGTTGATCCGGAAGCGAATT  Reverse3: ATAAGAATGCGGCCGCTCACAGACCGTAGACACT |
| LC3B  (K65W) | Forward: CGCGGATCCATGCCGTCGGAGAAGACC  Reverse: TAAGCGCCTTCTAATTATCCAGATGAGCTCACTCATGTT  Forward2: AACATGAGTGAGCTCATCTGGATAATTAGAAGGCGCTTA  Reverse3: ATAAGAATGCGGCCGCTTACACTGACAATTTCAT |
| LC3C  (K32Q, F33H) | Forward: CGCGGATCCATGCCGCCTCCACAGAAA  Reverse: CACCGGGATTTTGTTGGGGTGCTGTGCCCGGATTCCAGCAAC  Forward2: CCCAACAAAATCCCGGTG  Reverse3: ATAAGAATGCGGCCGCCTAGAGAGGATTGCAGGG |
| LC3C  (F33Y) | Forward: CGCGGATCCATGCCGCCTCCACAGAAA  Reverse: CACCGGGATTTTGTTGGGGTACTTTGCCCGGATTCCAGC  Forward2: GCTGGAATCCGGGCAAAGTACCCCAACAAAATCCCGGTG  Reverse3: ATAAGAATGCGGCCGCCTAGAGAGGATTGCAGGG |
| LC3C  (V26G) | Forward: CGCGGATCCATGCCGCCTCCACAGAAA  Reverse: CTTTGCCCGGATTCCAGCTCCTTCCTCTTGTCTGATTGC  Forward2: GCTGGAATCCGGGCAAAG  Reverse3: ATAAGAATGCGGCCGCCTAGAGAGGATTGCAGGG |
| RavZ or  RavZ(C258S) | Forward: AAGAGATCTATGAAAGGCAAGTTAACA  Reverse: GTAGGGCCCCTATTTTACCTTAATGCC |
| RavZ LIR(1-2) | Forward: GGTGAGCTCGCCACCATGATGAAAGGCAAGTTAACAGGT  Reverse: CGCGGTACCTGTAGTTTTGTCAAGCTCTGAGT |
| RavZ MT-  LIR(3) | Forward: ACAAGCTCGAGCCGGTACAGCTTTCTGAATTTA  Reverse: GTCGCGGCCGCCTACTATTTTACCTTAATGCCAC |
| RavZ CA | Forward: AAGAGATCTTCTATTTATCCTCCCGAA  Reverse: CACGCGTCGACCGTTAATGTTTTACCTTCAGTCAA |
| nLIR(Fy) | Forward: GCAGAATTCAGGCCACCGGACGACGCT  Reverse: CCCAAGCTTGGAGCCGGACTCCTGTAT |
| cLIR(Fy) | Forward: CACGCGTCGACAGGCCACCGGACGACGCT  Reverse: CACGCGAGCTCGGAGCCGGACTCCTGTAT |
| nLIR(TP[T]) | Forward: GCAGAATTCTTCGTGTCGGAGGAGGAT  Reverse: CCCAAGCTTGCTGGGTGGAGCCGCGTA |
| cLIR(TP[T]) | Forward: CACGCGAGCTCGCTGGGTGGAGCCGCGTA  Reverse: CACGCGTCGACTTCGTGTCGGAGGAGGAT |
| nLIR(St) | Forward: GCAGAATTCAACAGTCAGGACCGGGTT  Reverse: CCCAAGCTTCACACCAACATCCCCCCA |
| cLIR(St) | Forward: CACGCGTCGACAACAGTCAGGACCGGGTT  Reverse: CACGCGAGCTCCACACCAACATCCCCCCA |
| nLIR(4B[T]) | Forward: GCAGAATTCGAAAGATTCTTCGACTCA  Reverse: CCCAAGCTTAAGGGACAGGATTTCGGT |
| cLIR(4B[T]) | Forward: CACGCGTCGACGAAAGATTCTTCGACTCA  Reverse: CACGCGAGCTCAAGGGACAGGATTTCGGT |
| nLIR(Sp) or  (Sp[I]) | Forward: GCAGAATTCCTCCAATTTGAAATAGAC  Reverse: CCCAAGCTTATAATCACCATAACGCAT |
| cLIR(Sp) or  (Sp[I]) | Forward: CACGCGTCGACCTCCAATTTGAAATAGAC  Reverse: CACGCGAGCTCATAATCACCATAACGCAT |
